# Tailoring the Structure and Surface Chemistry of High-Loading Ni-Metakaolin Catalysts Prepared by Melt Infiltration for CO_2_ Methanation

**DOI:** 10.3390/molecules31162847

**Published:** 2026-08-14

**Authors:** Agnieszka Szymaszek-Wawryca, Michał Szymaszek, Robert Kosydar, Dorota Duraczyńska, Monika Motak

**Affiliations:** 1 Faculty of Energy and Fuels, AGH University of Krakow, al. A. Mickiewicza 30, 30-059 Krakow, Poland; szymaszek@student.agh.edu.pl (M.S.); motakm@agh.edu.pl (M.M.); 2 Jerzy Haber Institute of Catalysis and Surface Chemistry, Polish Academy of Sciences, Niezapominajek 8, 30-239 Krakow, Poland; robert.kosydar@ikifp.edu.pl (R.K.); dorota.duraczynska@ikifp.edu.pl (D.D.)

**Keywords:** CO_2_ methanation, kaolin, nickel, ceria, alkaline earth metals

## Abstract

CO_2_ methanation is a promising power-to-gas technology that enables the conversion of carbon dioxide into methane. However, the development of efficient catalysts based on naturally abundant and inexpensive support remains an important challenge. In this work, metakaolin from natural kaolin was investigated as a novel support for high-loading (30 wt.%) Ni catalysts prepared using a melt infiltration method. The influence of CeO_2_ and alkaline earth metal oxides (MgO, CaO) on the physicochemical properties and catalytic performance was systematically evaluated. It was evidenced that CeO_2_ improved NiO reducibility, whereas MgO and CaO promoted Ni^0^ dispersion and modified textural and surface properties. In particular, Mg addition increased the *S_BET_* from 23 to 39 m^2^/g and the total pore volume from 0.06 to 0.17 cm^3^/g compared with the Ni-MK sample. The promoted catalysts exhibited enhanced low-temperature activity and reached approximately 80% CO_2_ conversion at 400 °C, close to thermodynamic equilibrium, maintaining CH_4_ selectivity above 97%. Stable catalytic performance was preserved during 24 h time-on-stream tests. The results demonstrate that metakaolin is a promising sustainable support for Ni CO_2_ methanation catalysts and that melt infiltration provides a simple and effective preparation route for obtaining high nickel loading.

## 1. Introduction

Anthropogenic carbon dioxide (CO_2_) emissions from fossil fuel combustion have been a major driver of global warming since the mid-20th century [1]. Increasing CO_2_ concentrations contribute to extreme weather events, sea-level rise, and risks to food and water security, with projections estimating levels of ca. 790 ppm by 2100 and a global temperature increase of ca. 3.8 °C [2]. These challenges have accelerated the development of carbon capture, storage, and utilization (CCUS) strategies focused on converting CO_2_ into value-added products [3]. In this context, the catalytic hydrogenation of CO_2_ to methane (CH_4_) has emerged as a promising route for both greenhouse gas mitigation and energy production [4,5].

Methanation, also known as the Sabatier reaction (CO_2_ + 2H_2_ ⇔ CH_4_ + 2H_2_O, ∆*H*_298K_ = −165 kJ/mol), typically operates between 200 and 450 °C, depending on catalyst properties and reaction conditions. Although thermodynamically favorable, the reaction is kinetically complex due to the high stability of CO_2_, resulting in limited activity below 350 °C [6,7]. Additionally, competing reactions such as the reverse water–gas shift (RWGS) and carbon formation via the Boudouard reaction negatively affect methane selectivity and catalyst stability [8]. These challenges have driven the development of more efficient and robust catalysts, with nickel-based systems receiving particular attention, due to their favorable activity-cost balance [9,10]. However, the narrow operating temperature window of Ni-catalysts has motivated the design of multi-metallic systems, especially those promoted with lanthanide or alkaline earth metals [11,12,13,14,15].

Cerium oxide is among the most effective rare-earth promoters for CO_2_ methanation catalysts due to its redox properties associated with the Ce^4+^/Ce^3+^ couple, oxygen vacancy formation, and moderate basicity [16,17,18]. Even small amounts of Ce have been shown to enhance the catalytic performance of Ni-based systems [13,16,17,18,19]. Gac et al. [17] reported that Ce modification improves Ni dispersion and increases the number of CO_2_ adsorption sites during methanation. Jiang and Lian [18] showed that small amounts of CeO_2_ promote the formation of Ni-O-Ce interfacial sites on Al_2_O_3_-supported catalysts, increasing surface basicity and facilitating the formation and decomposition of formate intermediates, which enhances CO and CH_4_ production. Aimdate et al. [20] demonstrated that co-deposition of Ni and Ce on metakaolin increases specific surface area and improves CH_4_ yield, attributing the promotional effect of Ce to oxygen vacancy formation and enhanced H_2_ activation on Ni sites.

In parallel, alkali and alkaline earth metals are widely employed as promoters in CO_2_ methanation catalysts, which is related to their ability to enhance surface basicity, improve CO_2_ adsorption, and suppress sintering of the active phase [12]. Compared to alkali metals, Mg and Ca form thermally stable oxides that provide stronger basic sites and higher resistance to volatilization [12,21]. Jiang et al. [22] reported that Na blocked Ni active sites, whereas Mg strengthened metal–support interactions, limiting CO_2_ adsorption. In contrast, rare-earth promoters enhanced low-temperature activity by creating oxygen vacancies and modifying Ni-support interactions. However, the synergistic effect of alkaline earth metals and Ce remains poorly understood.

**Figure 1:**
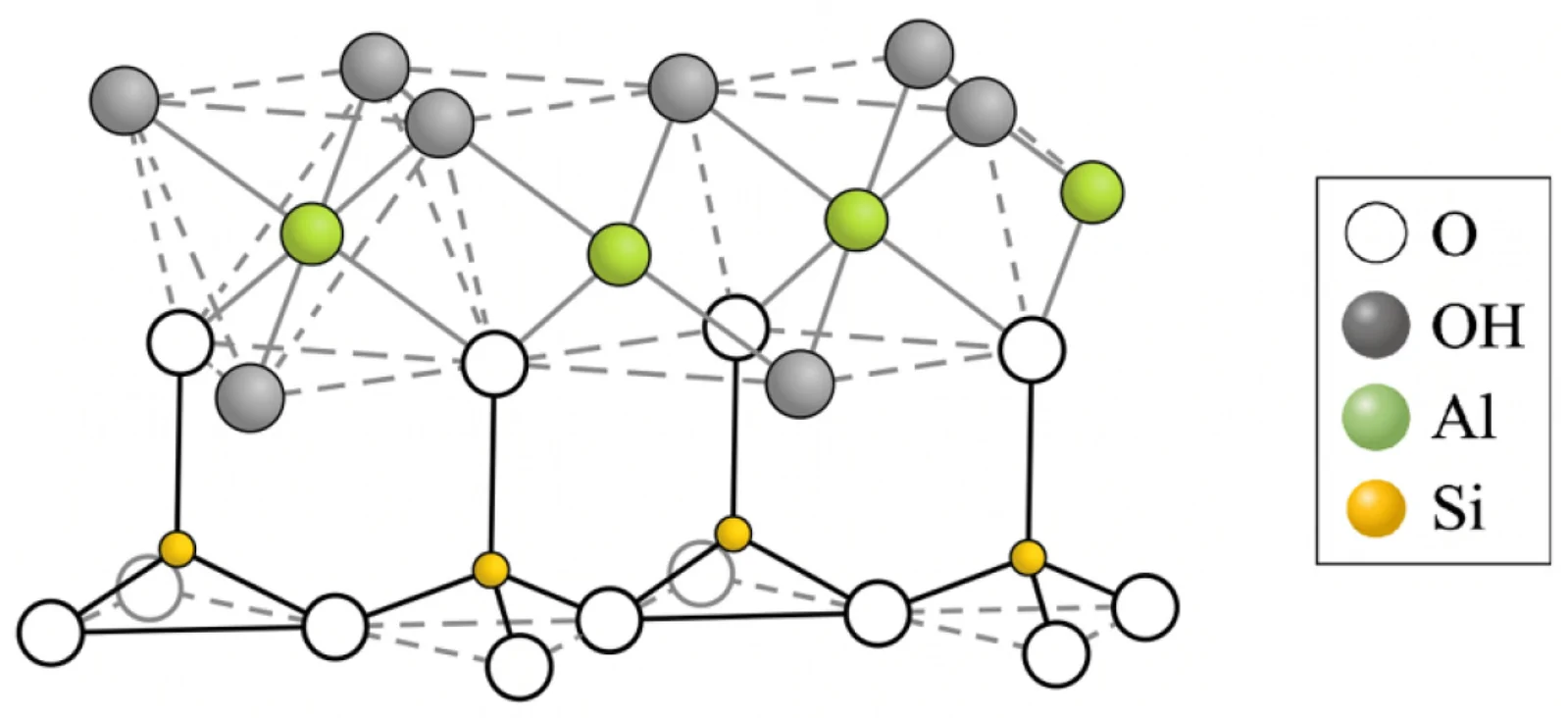
Schematic representation of the layered structure of kaolinite (own work based on [33]).

Beyond the active phase and promoters, catalyst performance is strongly influenced by the choice of support, which governs dispersion, reducibility, and CO_2_ activation [7,23]. Conventional oxide supports, such as SiO_2_, Al_2_O_3_, TiO_2_, and ZrO_2_ are effective; however, they are often associated with high costs and environmental burden [6,24,25,26]. As a result, natural clays have emerged as attractive alternatives, due to their abundance, low cost, and tunable surface properties. Kaolin, a layered aluminosilicate, is particularly promising owing to its chemical composition which may further benefit CO_2_ methanation [20,27]. The mineral is widely used in the construction [28], paper [29], and ceramic industries [30]. Chemically, kaolin mainly consists of 1:1 nanolayered kaolinite (Al_2_Si_2_O_5_(OH)_4_, in which each individual layer is composed of a tetrahedral sheet (SiO_4_) and an octahedral sheet (Al(O,OH)_6_). The sheets share a common plane of oxygens and hydroxyl groups and are connected by strong covalent bonds [31]. Furthermore, the layers are stabilized by hydrogen bonds formed from oxygens of Si tetrahedra and hydroxyl groups from Al octahedra, from the adjacent layer. The presence of these bonds prevents big or non-polar molecules from grafting or intercalating [32]. The structure of kaolinite is schematically presented in Figure 1.

Due to its favorable physicochemical properties, kaolin is a promising sustainable support for novel CO_2_ methanation catalysts. Thermal transformation into amorphous metakaolin produces a support that effectively suppresses nickel particle sintering, thereby enhancing catalyst stability [34,35,36,37,38]. Despite these advantages, metakaolin-supported catalysts for CO_2_ methanation have received relatively little attention. Previous studies demonstrated favorable Ni dispersion and strong interactions with the aluminosilicate matrix [20,39]; however, the influence of metal promoters on the structure–performance relationships of these catalysts remains insufficiently understood. Furthermore, achieving high Ni loadings (30 wt.%) while maintaining highly dispersed nickel nanoparticles remains a significant challenge, particularly for low-surface-area supports.

Recently, the melt infiltration (MI) method has emerged as a scalable approach for preparing catalysts with high metal loadings, while maintaining excellent dispersion [40,41,42]. Its successful application to porous supports, such as SBA-15 and γ-Al_2_O_3_ demonstrates its potential for producing well-dispersed metallic nanoparticles. Given these advantages, the application of MI for the preparation of metakaolin-supported methanation catalysts appears highly promising, yet has received limited attention.

In this work, the effects of cerium and selected alkaline earth metals (Mg and Ca) on the performance of Ni/metakaolin catalysts for CO_2_ methanation were systematically investigated. Catalysts with high Ni loadings were prepared via a one-step melt infiltration method. By establishing structure–performance relationships, this study identifies the most effective promoter system and demonstrates the potential of melt infiltration as a simple, scalable, and sustainable route for the fabrication of efficient CO_2_ methanation catalysts based on abundant mineral resources.

## 2. Results

### 2.1. Characterization of the Catalysts

#### 2.1.1. Crystallinity and Phase Composition Determined by X-Ray Diffraction (XRD)

**Figure 2:**
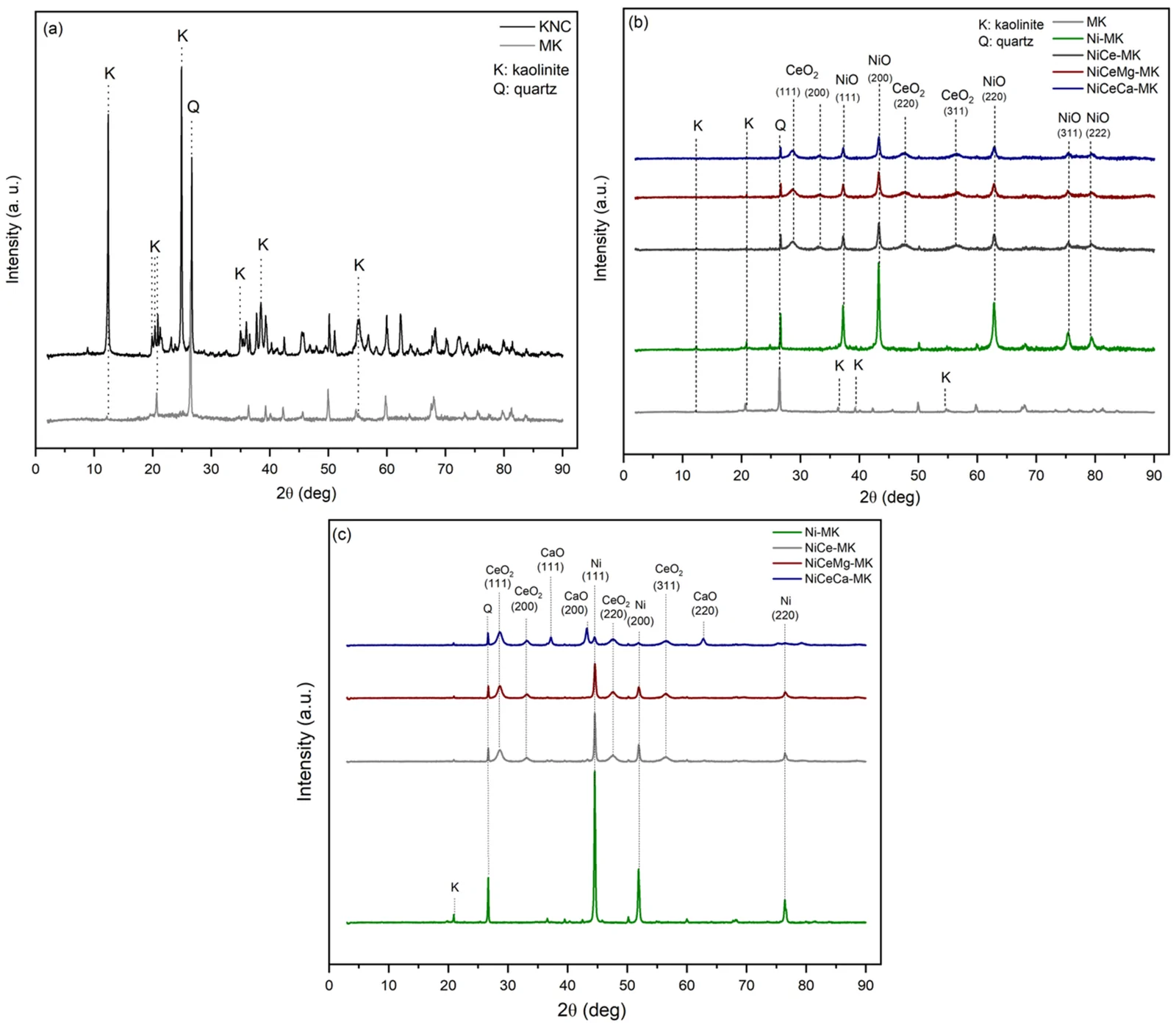
XRD patterns obtained for (**a**) kaolin (KNC) and metakaolin (MK) (**b**) as-prepared catalysts (**c**) reduced catalysts.

The XRD patterns recorded for the support before and after dehydroxylation (Figure 2a) clearly demonstrate transformation from kaolin into metakaolin. The obtained results indicated that the raw clay is abundant in kaolinite (2*θ* = 12.4 (001), 19.9 (020), 20.4 ($\bar{1}$10), 20.9 (110), 25.1 (002), 34.9 (130), 38.5 ($\bar{1}$31), and 55.2° (300)) [43,44] and quartz (2*θ* = 26.6, consistent with JCPDS card no. 05-0490) [20]. The diffractograms confirmed that thermal treatment of kaolin resulted in the collapse of the structure into amorphous, metakaolin phase, due to the removal of hydroxyl groups and release of interlayer water molecules [39,43]. As reported in the literature [20,39], the metakaolin phase formed during calcination of kaolinite should exhibit only one, broad diffraction peak within 2*θ* of 15–35 due to the presence of SiO_2_. However, some of the kaolinite-characteristic diffraction signals are still present in the XRD pattern of the MK sample, most likely due to relatively low temperature during thermal activation [39].

Figure 2b illustrates XRD patterns of the as-prepared catalysts. The low-intensity reflections assigned to kaolinite and quartz remained unchanged after metal incorporation, indicating that the melt infiltration method did not alter the structure of the metakaolin support. The diffraction peaks appearing at 2*θ* = 37.3, 43.2, 62.9, 75.3, 79.4°, with the diffracting planes of (111), (200), (220), (311), (222), accordingly, correspond to the cubic NiO (JCPDS card no. 47-1049) [45]. Furthermore, all ceria-modified samples exhibited diffraction maxima located at 2*θ* = 28.7 (111), 33.3 (200), 47.7 (220), and 56.6° (311), corresponding to the cubic CeO_2_ (fluorite structure, in line with JCPDS card no. 34-0394) [46]. No diffraction peaks assigned to MgO, CaO, or their compounds were observed, likely because of their high dispersion or overlap with the reflections of the support, NiO, or CeO_2_.

Figure 2c shows the XRD patterns of the reduced catalysts. Activation in H_2_ resulted in the formation of metallic Ni^0^, with characteristic reflections at 2*θ* = 44.3 (111), 51.7 (200), and 76.1° (220) (JCPDS card no. 03-1051) [47]. The position of the diffraction maxima attributed to CeO_2_ remained unchanged. For the as-prepared catalysts, no MgO reflections were detected, suggesting its high dispersion or amorphous character. In contrast, weak signals assigned to CaO (2*θ* = 37.2 (111), 43.3 (200), and 62.8 (220) (JCPDS card no. 37-1497) were observed for NiCeCa-MK. The crystallite sizes of NiO, CeO_2_, and metallic Ni^0^ in the reduced and spent catalysts were estimated using the Scherrer Equation (1) from the most intense (111) diffraction peak. As shown in Table 1, NiO crystallites ranged from 17 to 24 nm, whereas CeO_2_ crystallites remained small (6–7 nm) regardless of catalyst composition. The addition of ceria slightly decreased the NiO crystallite size, in agreement with previous reports [18,48]. The effect became more pronounced after co-promotion with MgO or CaO, with the smallest NiO crystallites (17 nm) observed for NiCeMg-MK. The lower intensity of the NiO reflections further indicates improved dispersion of the active phase, which likely contributed to the enhanced catalytic performance. Reduction resulted in a slight increase in Ni crystallite size, indicating limited sintering. Nevertheless, the beneficial effect of alkaline earth promoters on Ni crystallite size was preserved after reduction. After one catalytic cycle and a 24 h stability test at 350 °C, the Ni crystallite sizes remained comparable to those of the reduced catalysts, except for NiCeCa-MK, where they increased from 25 to 30 nm. This moderate growth may be attributed to the higher crystallinity of the metallic phase, resulting in a greater tendency toward agglomeration under reaction conditions.

#### 2.1.2. Textural Properties Determined by N_2_ Adsorption at 77 K

**Figure 3:**
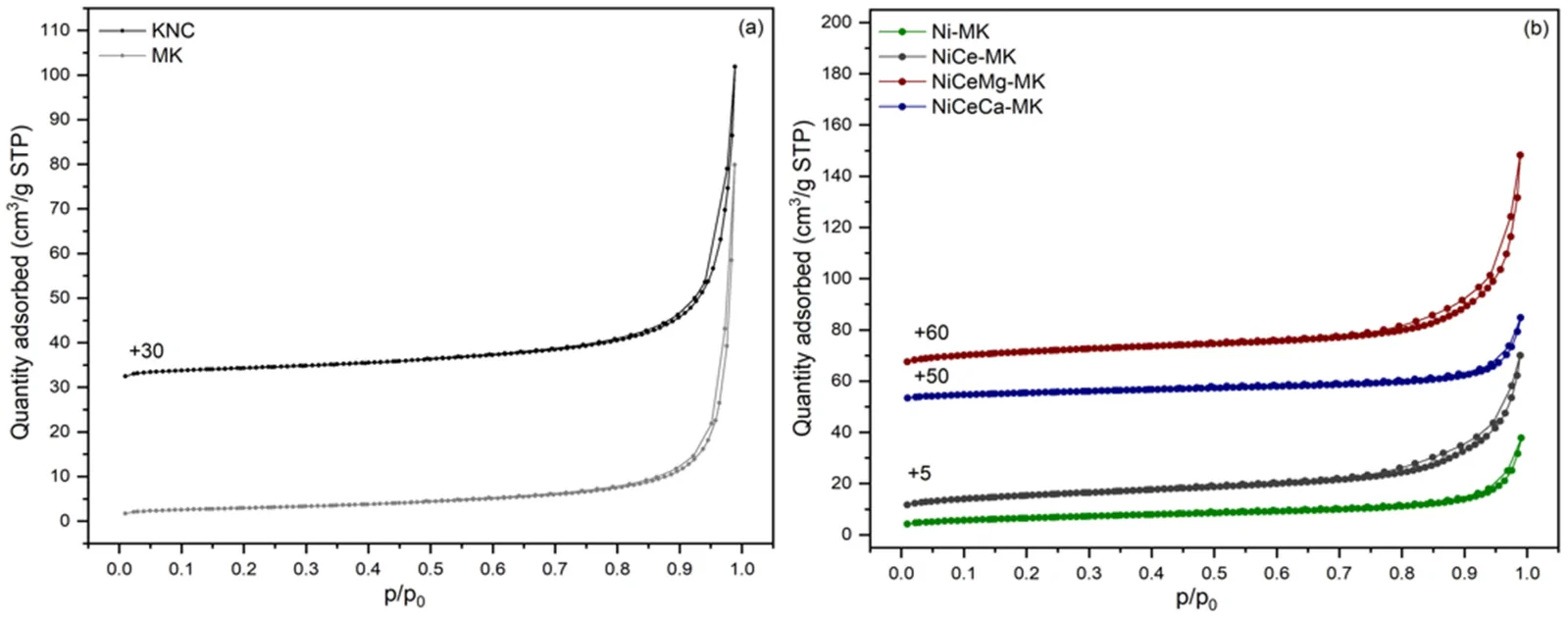
N_2_ sorption isotherms of (**a**) kaolin (KNC) and metakaolin (MK), (**b**) the catalysts (for better visibility, the adsorption–desorption branches are shifted by the values given in the figures).

The structural and textural properties were evaluated by N_2_ sorption at 77 K. The adsorption–desorption isotherms of the support and catalysts are presented in Figure 3a,b, respectively. The N_2_ adsorption–desorption isotherms of kaolin (KNC) and metakaolin (MK) resemble type III according to the IUPAC classification, indicating weak adsorbate-adsorbent interactions [49,50]. A narrow H3 hysteresis loop at high relative pressures suggests the presence of slit-shaped mesopores formed by the aggregation of plate-like particles, while the low N_2_ uptake at low *p*/*p*_0_ confirms the negligible contribution of micropores. The differences between KNC and MK reflect the structural rearrangement induced by thermal treatment.

As shown in Figure 3b, all catalysts exhibited combined type III/IV isotherms with H3 hysteresis loops. However, differences in N_2_ uptake reflected changes in textural properties. The highest adsorption, observed for NiCeMg-MK, indicates a synergistic effect of CeO_2_ and MgO, resulting in the largest specific surface area and pore volume (Table 2). This behavior is consistent with the pore-widening effect of MgO [51], while CeO_2_ has been reported to prevent pore blockage by NiO species and preserve mesoporosity [20].

The textural parameters of the support and as-prepared catalysts are summarized in Table 2. Calcination of kaolin decreased the specific surface area while increasing the average pore size, whereas the total pore volume remained nearly unchanged. These changes are attributed to the transformation of crystalline kaolin into amorphous metakaolin, which induces structural rearrangement and partial sintering of the aluminosilicate layers. Consequently, smaller pores coalesced into larger mesopores, reducing the available surface area without significantly affecting the total pore volume. Similar trends have been reported by Liu et al. [39] and are consistent with the XRD and SEM results.

The textural properties of the catalysts strongly depended on the composition of the active phase, whereas the average pore size remained nearly unchanged. Compared with MK, Ni-MK exhibited a higher *S_BET_*, most likely due to gas evolution during calcination and the formation of additional mesoporosity. The increased *S_ext_* can be attributed to well-dispersed NiO nanoparticles, while the lower *V_total_* suggests partial pore filling by NiO species. Compared with Ni-MK, the addition of ceria further increased *S_BET_* and slightly increased *S_micro_*, in line with literature reports attributing this effect to improved NiO dispersion and the porous nature of CeO_2_ [13,20]. In contrast, Mg and Ca affected the textural properties differently. Mg increased *V_micro_* and *V_total_*, whereas Ca reduced *S_BET_* and *S_ext_*, as well as pore volume, likely due to greater pore occupation by the active phase or the formation of small amounts of calcium aluminate, CaAl_2_O_4_, as also reported in the literature [11,52].

**Figure 4:**
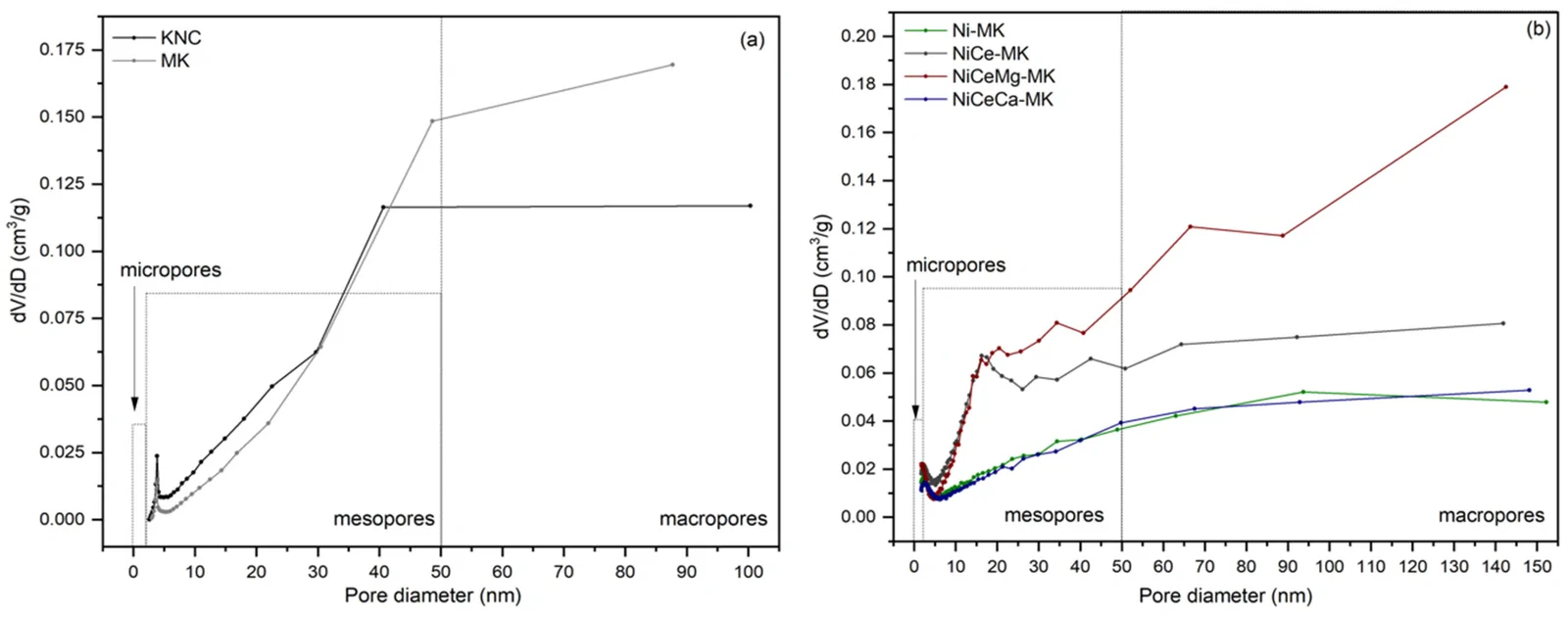
Pore size distributions of (**a**) kaolin (KNC) and metakaolin (MK) and (**b**) the catalysts determined from N_2_ sorption at 77 K.

Figure 4 shows that the pore size distribution (*PSD*) of the support spans approximately 1–100 nm, extending up to 150 nm for the catalysts. All materials exhibited a predominantly mesoporous structure with a minor contribution of macropores, particularly after metal incorporation. The main mesopore peak shifted from ~40 nm for kaolin (Figure 4a) to ~48 nm for metakaolin, indicating pore widening during calcination. This behavior is attributed to the release of structural water and the accompanying rearrangement of the aluminosilicate framework, resulting in enhanced mesoporosity. Figure 4b shows that metal incorporation markedly altered the pore size distribution compared to metakaolin. All catalysts exhibited broad PSD curves, indicating the formation of pores with a wide range of sizes. Ni-MK showed a maximum centered at 50–60 nm, characteristic of meso- and small macropores. NiCe-MK and NiCeMg-MK exhibited the highest pore volumes, particularly in the macropore range, suggesting that CeO_2_, especially in combination with MgO, promotes pore enlargement, consistent with previous reports [51,53]. In contrast, Ca incorporation reduced the pore volume, indicating partial pore blocking.

#### 2.1.3. Morphology and Elemental Distribution Analyzed by SEM/EDS

**Figure 5:**
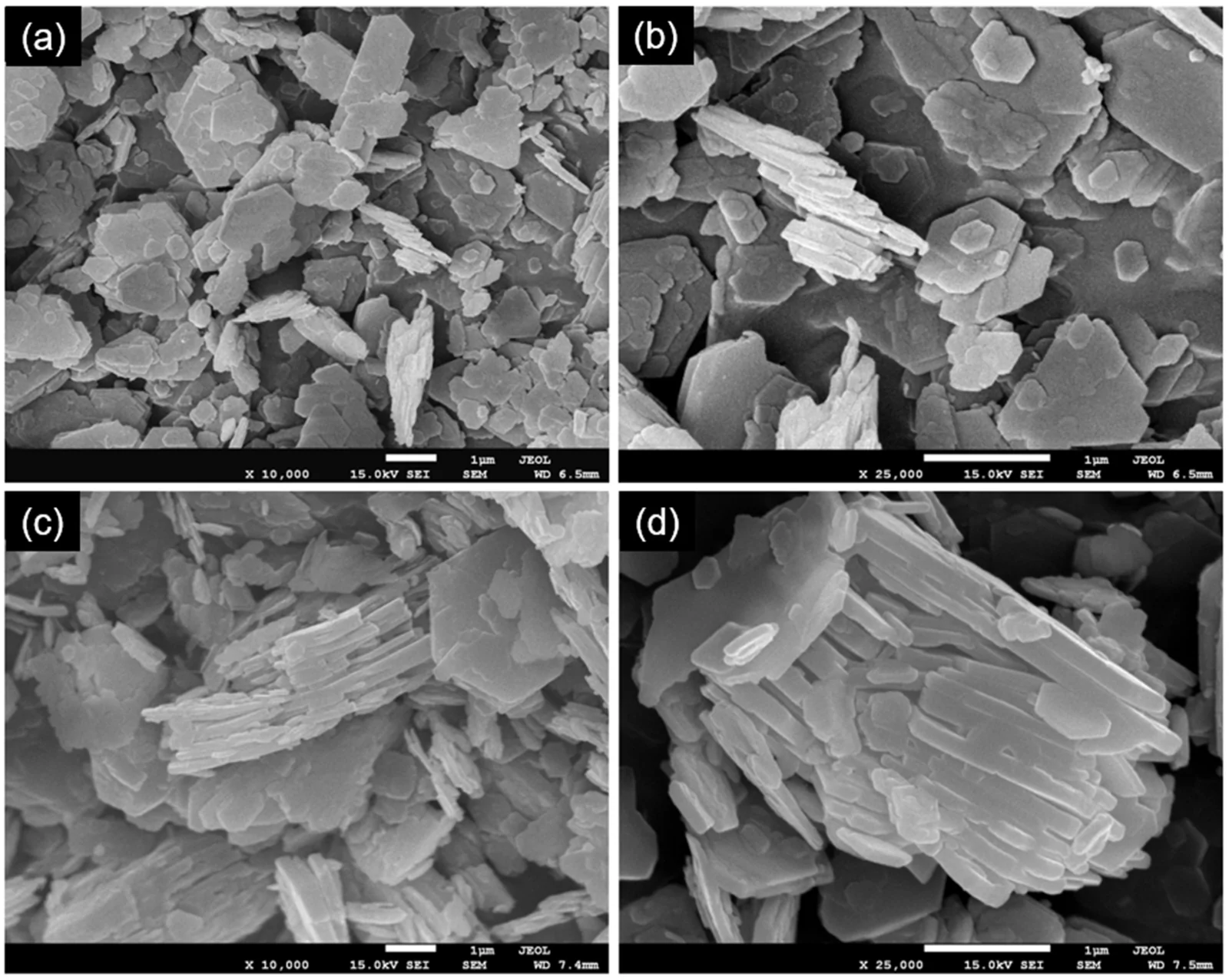
SEM micrographs of kaolin (**a**,**b**) and metakaolin (**c**,**d**) obtained at low (×10,000; **a**,**c**) and high (×25,000; **b**,**d**) magnification.

The morphology and elemental distribution were analyzed using scanning electron microscopy equipped with energy-dispersive X-ray detector (SEM/EDS). Figure 5 presents the images recorded for kaolin and metakaolin. Inspection of these micrographs revealed that both materials are characterized by agglomerated, plate-like particles with irregular shapes and dimensions. The smooth and clean surface is typical for non-porous clays, which corroborates with the N_2_ sorption data, confirming low specific surface area and negligible porosity.

**Figure 6:**
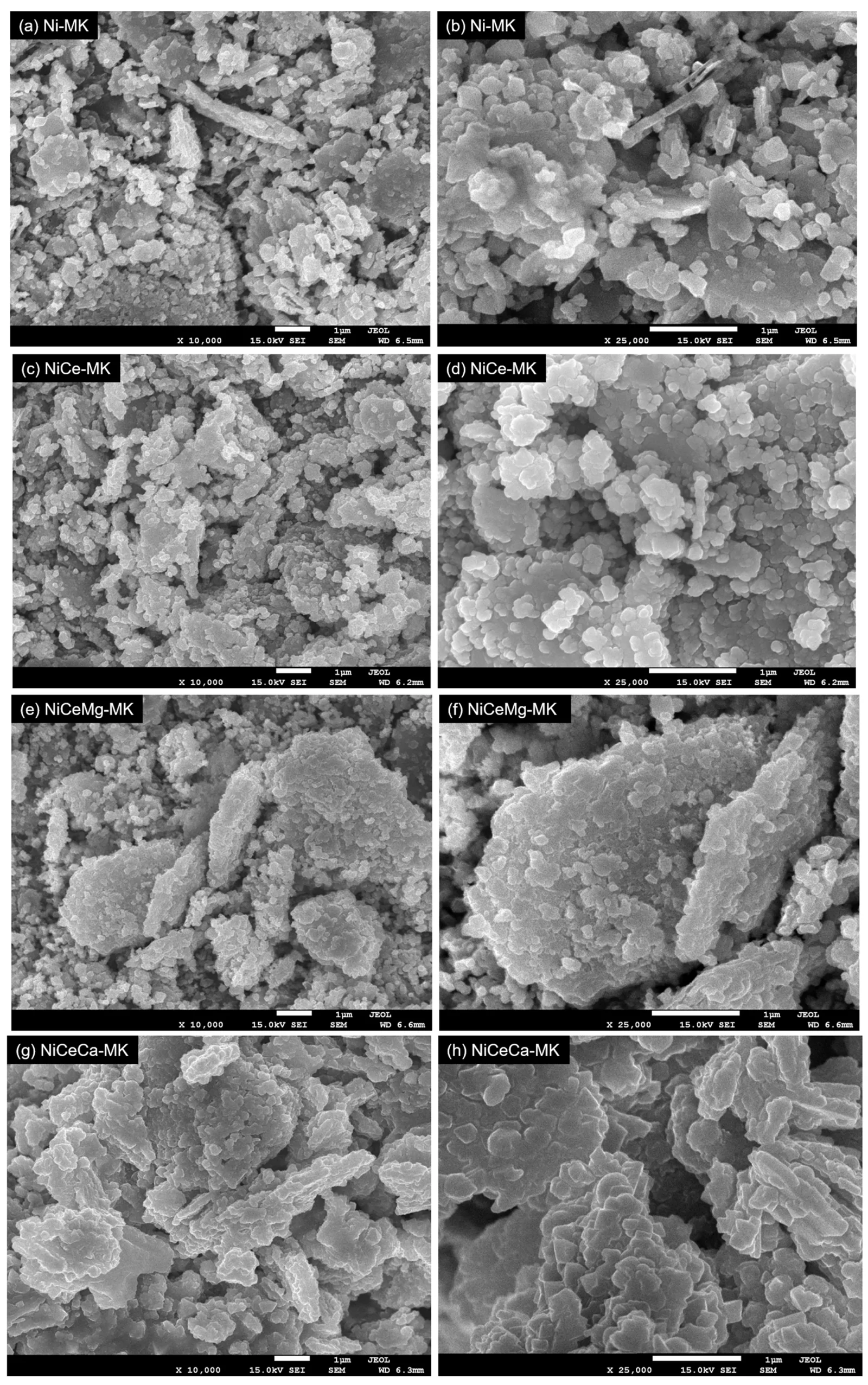
SEM images recorded for the as-synthesized catalysts at (**a**,**c**,**e**,**g**) lower (×10,000) and (**b**,**d**,**f**,**h**) higher (×25,000) magnification (the codes of the samples are visible in the micrographs).

From SEM images of the as-prepared catalysts (Figure 6), it becomes apparent that the metal oxides deposited via the melt infiltration method are well dispersed across the surface of metakaolin. Simultaneous introduction of nickel oxide and ceria moderately reduced the particle size, with the effect becoming more pronounced upon joint introduction of CeO_2_ and alkaline earth metal oxides. Strikingly, this behavior did not depend on the identity of the Group II element.

**Figure 7:**
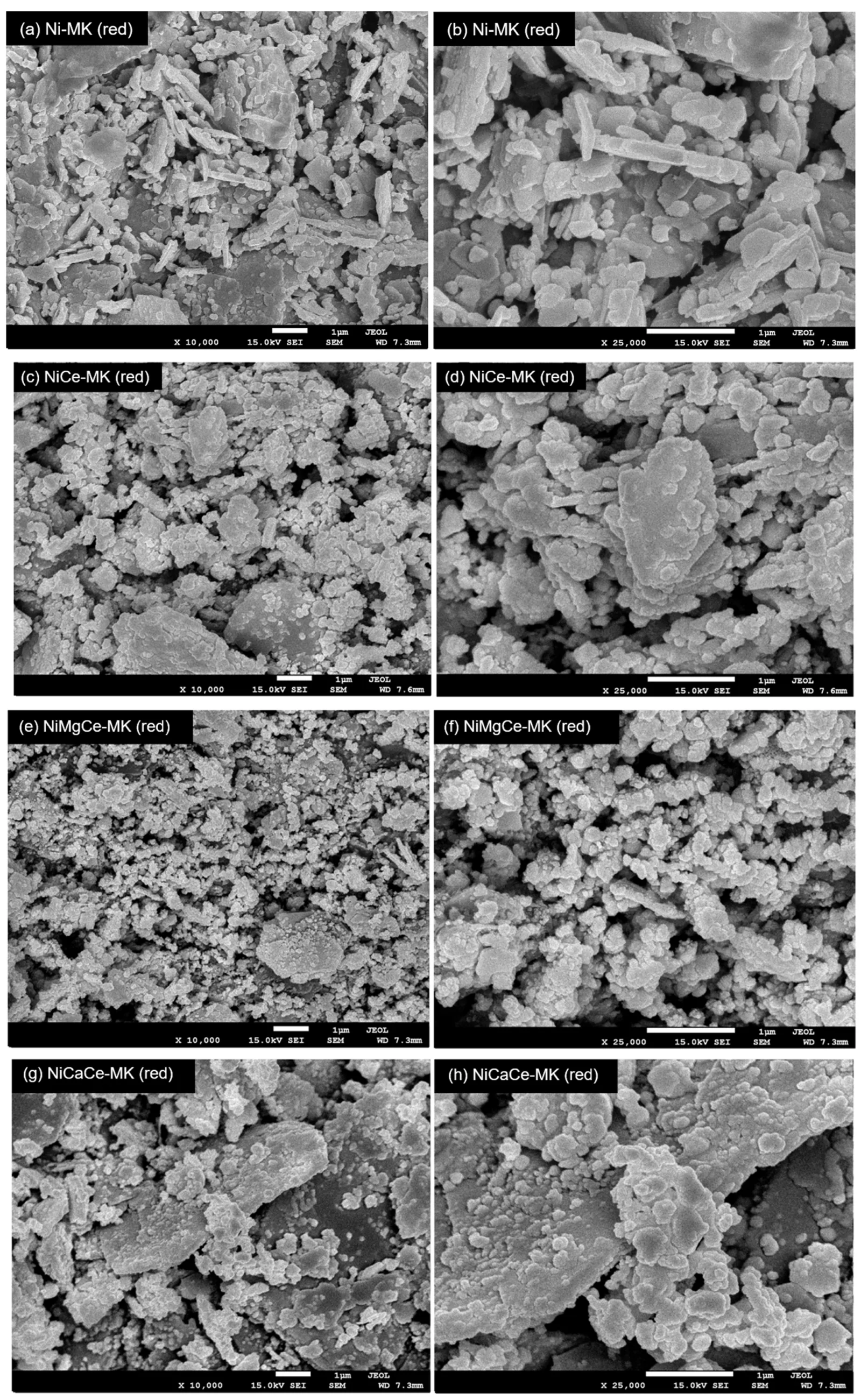
SEM images recorded for the reduced catalysts at (**a**,**c**,**e**,**g**) lower (×10,000) and (**b**,**d**,**f**,**h**) higher (×25,000) magnification (the codes of the samples are visible in the micrographs).

SEM micrographs of the reduced catalysts (Figure 7) indicated that pre-treatment with H_2_ did not influence the plate-like morphology of the aluminosilicate. Similarly to the as-prepared samples, the metallic phase is uniformly distributed and the effect was even stronger while CeO_2_, and MgO or CaO were simultaneously present. Overall, the morphological outcomes are consistent with XRD results, further highlighting the crucial impact of CeO_2_, alkaline earth metal oxides and their co-existence on the particle dispersion.

The EDS analysis (Table 3) confirmed the expected elemental composition of all samples. Kaolin consisted mainly of Si, Al, and O, whereas metal incorporation introduced the corresponding active components. Minor deviations between the measured and nominal compositions can be attributed to the local nature of EDS analysis. Moreover, no microscale segregation of CeO_2_, MgO, or CaO was observed, indicating their uniform distribution and confirming the effectiveness of the melt infiltration method.

**Figure 8:**
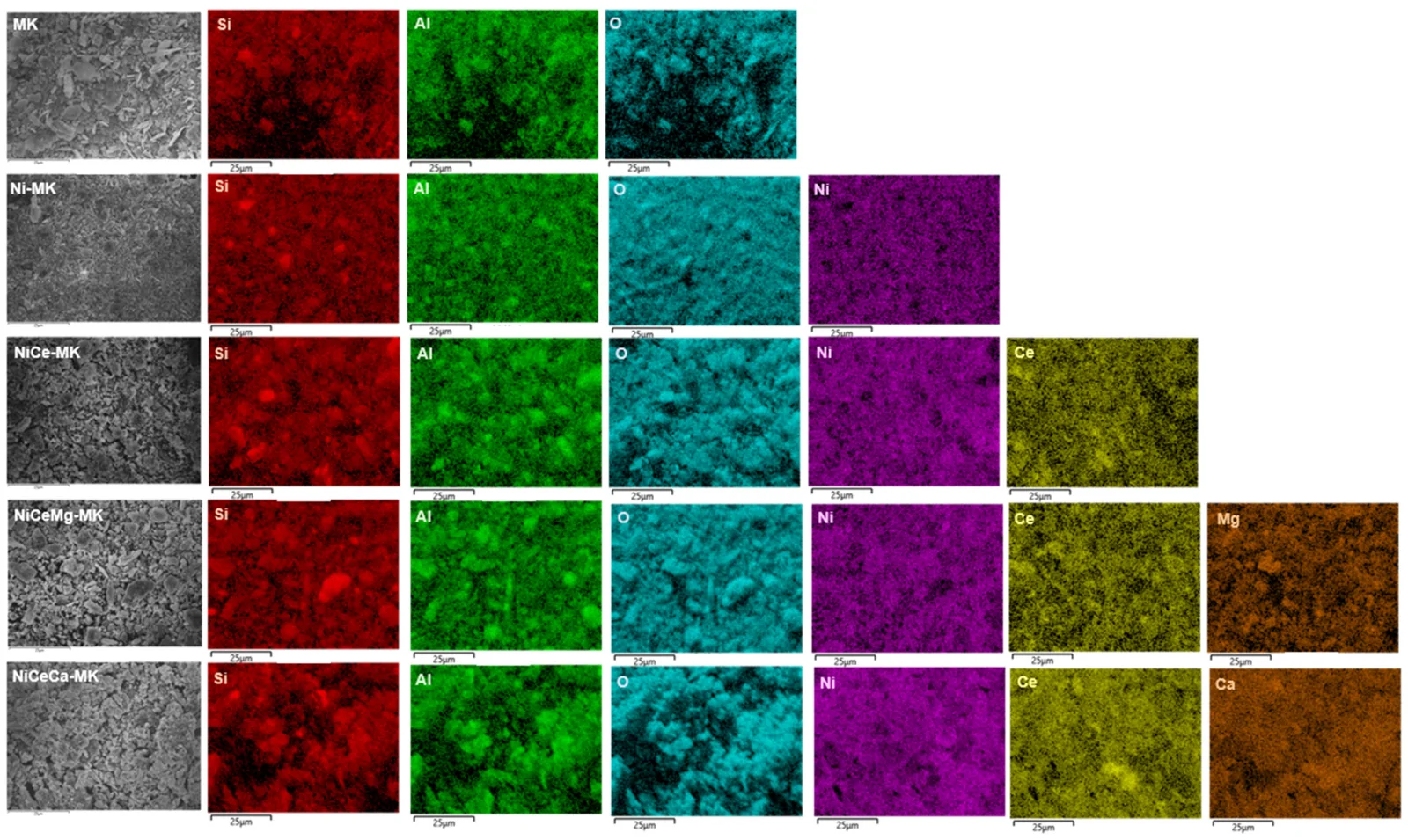
EDS elemental mappings showing the distribution of Ni, Ce, and alkaline earth metals (where present) in the as-prepared catalysts.

**Figure 9:**
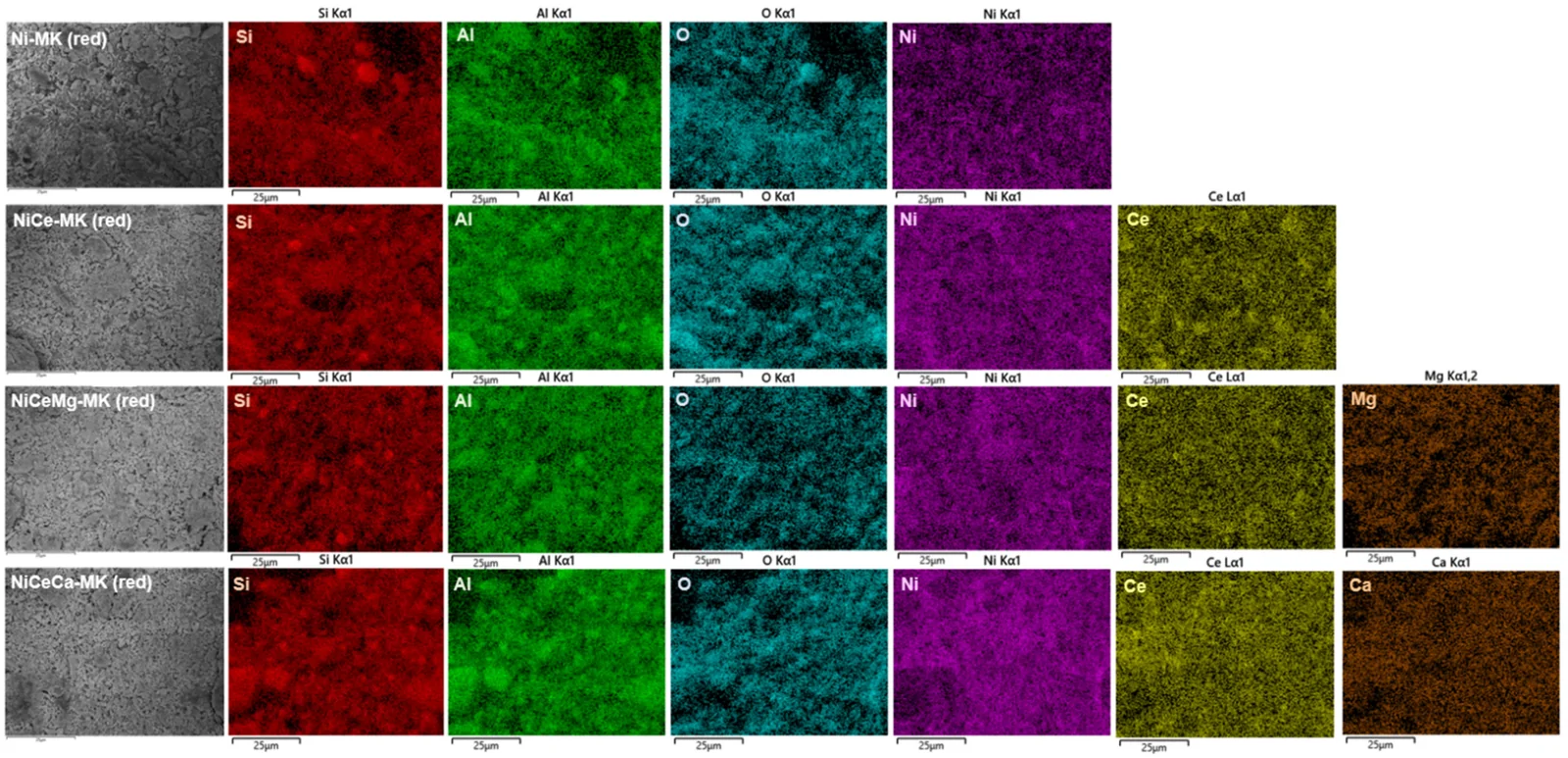
EDS elemental mappings showing the distribution of Ni, Ce, and alkaline earth metals (where present) in the reduced catalysts.

Figure 8 shows the elemental distribution obtained by EDS mapping. All metals were uniformly dispersed over the metakaolin surface, confirming the effectiveness of the melt infiltration method. The only exception was Mg, which exhibited slight local enrichment, suggesting limited MgO agglomeration. Nevertheless, no significant segregation of the active phase was observed. As shown in Figure 9, the reduced catalysts retained a similarly uniform elemental distribution, particularly for the Mg- and Ca-promoted samples.

#### 2.1.4. Temperature-Programmed Reduction with Hydrogen (H_2_-TPR)

**Figure 10:**
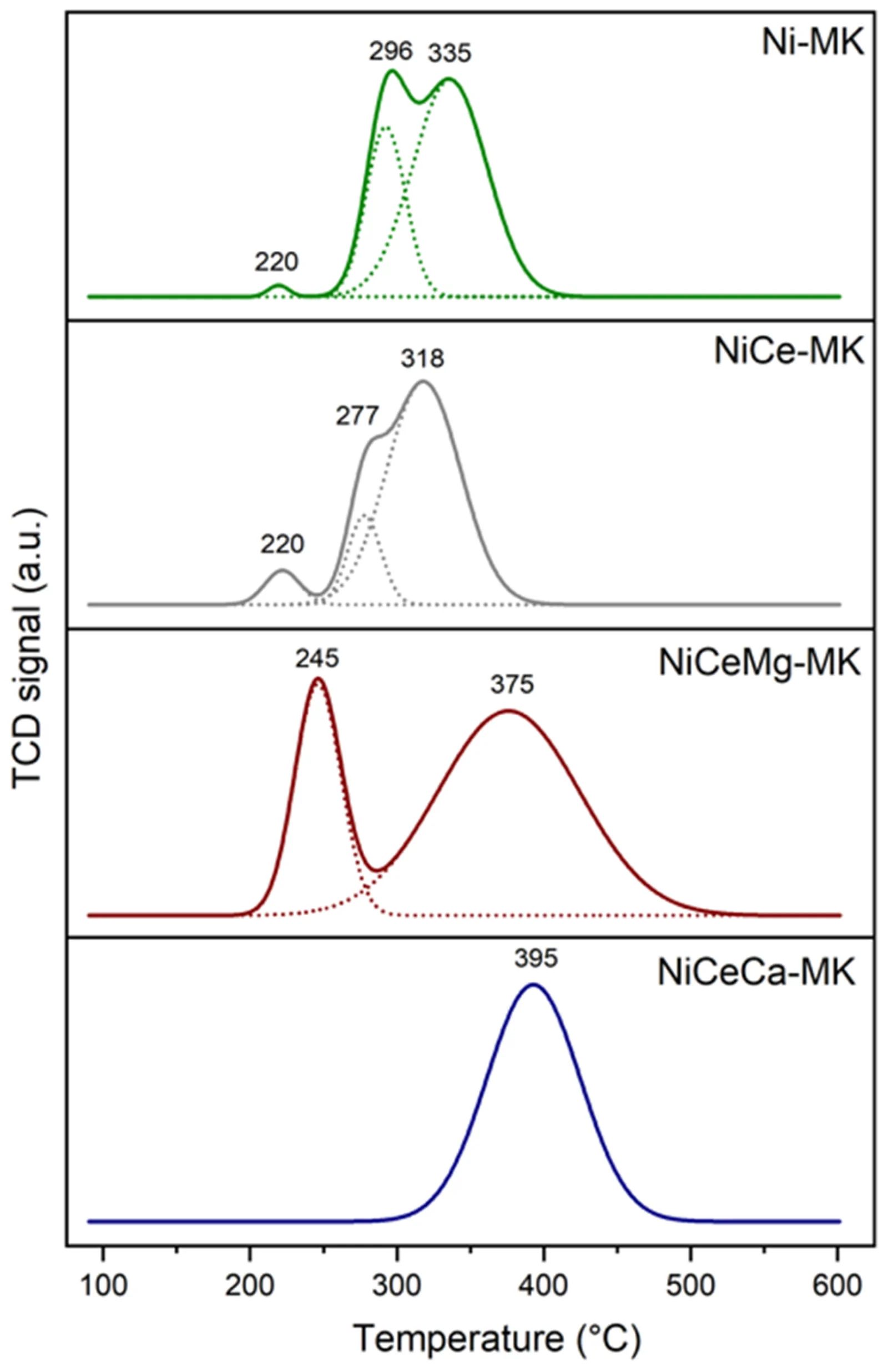
H_2_-TPR profiles obtained for the investigated catalysts.

H_2_-TPR was used to evaluate the reducibility of metal oxides and the effect of promoters on metal–support interactions. Figure 10 shows the normalized TPR patterns of the catalysts, which varied markedly with catalyst composition. In general, the profiles can be divided into three Gaussian-fitted regions corresponding to different NiO species and their location within the catalyst [14,54]. Highly dispersed NiO reduces at lower temperatures, whereas larger crystallites require higher reduction temperatures [54]. For all catalysts, the first region is centered below 400 °C and is assigned to the reduction of NiO species weakly interacting with the external surface of the aluminosilicate support [19]. However, the position and shape of these reduction features differ depending on the catalyst composition. The Ni-MK sample exhibited three peaks centered at 230, 285, and 324 °C, typical for the reduction of free NiO formed during thermal decomposition of Ni(NO_3_)_2_ [55]. The TPR profile of NiCe-MK showed that the addition of ceria shifted the reduction features toward lower temperatures. Zhou et al. [56] and Ibrahim et al. [57] attributed this effect to stronger NiO-CeO_2_ interactions than those between NiO and the support. Bacariza et al. [19] further related the enhanced reducibility to the high oxygen mobility of ceria. In addition, XRD analysis of the as-prepared catalysts indicated a slight decrease in the NiO crystallite size after CeO_2_ incorporation, which may also contribute to the improved reducibility [58]. The incorporation of alkaline earth metals markedly altered the reduction behavior of the samples. The NiCeMg-MK catalyst exhibited two well-defined reduction peaks centered at 225 and 346 °C, indicating the presence of two distinct NiO species. Compared with NiCe-MK, the low-temperature peak shifted to lower temperatures, whereas the second peak moved to higher temperatures, indicating the coexistence of readily reducible NiO species and NiO interacting more strongly with the support. Despite these stronger interactions, the reduction feature around 600 °C, characteristic of a Ni-Mg solid solution [59], was not observed. In contrast, the incorporation of Ca shifted the low-temperature reduction peak to 250 °C, indicating slightly stronger interactions than in the Mg-promoted catalyst. This behavior may be related to the formation of small amounts of calcium aluminate, CaAl_2_O_4_ (CaO + Al_2_O_3_ ⟶ CaAl_2_O_4_), which could partially hinder H_2_ access to NiO particles, as previously reported for mesoporous catalytic systems [60]. The sharp reduction peak centered at 322 °C further suggests stabilization of Ni species through modification of the Al-O environment of the metakaolin support [61]. The reduction temperatures of both Mg- and Ca-promoted catalysts remained lower than those reported previously for similar systems [14,59], demonstrating the beneficial effect of the selected promoter composition on catalyst reducibility.

Quantitative hydrogen consumption, including the total H2 uptake and the relative contributions of the low- and high-temperature reduction events, is summarized in Table 4. The total hydrogen consumption ranged from 4.15 to 5.23 mmol/g, indicating comparable overall reducibility among the catalysts. For all samples, hydrogen consumption was dominated by the higher-temperature reduction events, suggesting that most NiO species interacted moderately with the support. Although the addition of promoters generally shifted the reduction features toward lower temperatures (Figure 10), the total hydrogen uptake varied among the catalysts, reflecting differences in the amount of reducible Ni species. Ni-MK exhibited the highest H_2_ consumption (5.23 mmol/g), indicating the largest fraction of reducible NiO. In contrast, NiCe-MK and NiCeMg-MK showed slightly lower hydrogen uptake, suggesting a higher proportion of NiO species interacting more strongly with the support. NiCeCa-MK displayed the highest contribution from the low-temperature reduction event (8.2%), indicating a greater fraction of readily reducible NiO species despite its slightly higher reduction temperature.

#### 2.1.5. Temperature-Programmed Desorption of CO_2_ (CO_2_-TPD)

The strength and distribution of the basic sites on the reduced catalysts were investigated by CO_2_ temperature-programmed desorption (CO_2_-TPD). Generally, the strength of the basic sites is reflected by the temperature at which CO_2_ desorption occurs. The desorption peaks are commonly classified into three temperature regions corresponding to weak (below 250 °C), medium (250–550 °C), and strong (above 550 °C) basic sites [62]. These sites are generally assigned to surface hydroxyl groups (Brönsted basic sites), metal–oxygen pairs (Lewis basic sites), and low-coordinated surface O^2−^ anions, respectively [63,64]. Since CO_2_ is one of the reactants in the methanation reaction, the CO_2_-TPD profiles also provide valuable information on its adsorption and activation over the catalyst surface. It is generally accepted that CO_2_ adsorption and activation occur predominantly on the support, whereas metallic Ni is mainly responsible for hydrogen activation [65]. Therefore, the incorporation of CeO_2_ and alkaline earth promoters is expected to modify the concentration, strength, and nature of the surface basic sites. Additionally, the low electronegativity of the promoting metals generates basic behavior of the catalysts [11].

**Figure 11:**
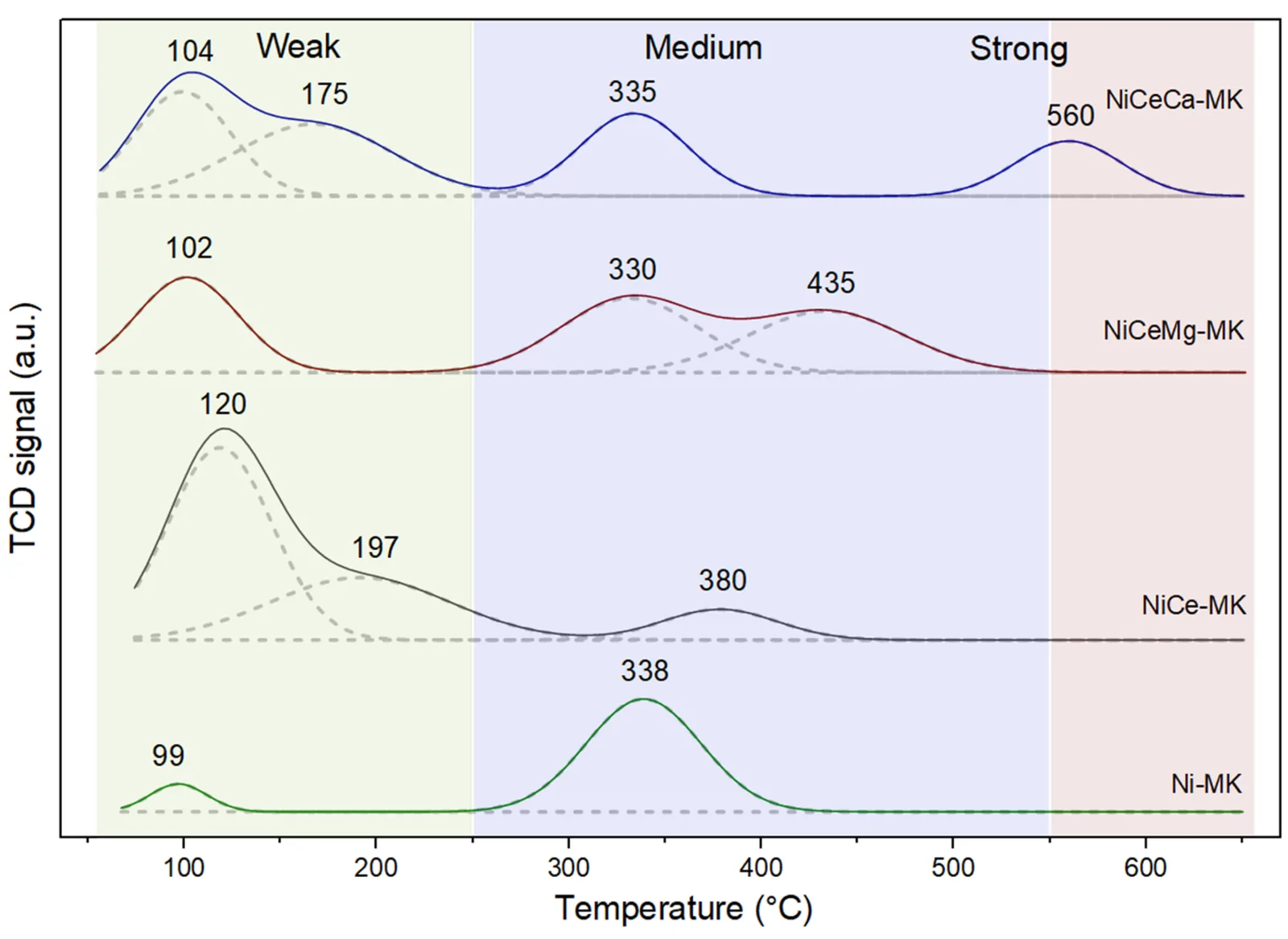
CO_2_-TPD profiles obtained for the reduced catalysts.

As shown in Figure 11, all catalysts exhibit weak and medium-strength basic sites, whereas strong basic sites are detected only for NiCeCa-MK. The promoter composition markedly influences both the distribution and the strength of the basic sites, indicating significant changes in the interaction of the catalyst surface with CO_2_. In the low-temperature region, Ni-MK exhibits only one weak desorption peak centered at 99 °C. The incorporation of CeO_2_ increases the strength of the weak basic sites, as evidenced by the higher desorption temperature (120 °C), and simultaneously leads to the formation of an additional population of weak basic sites, evidenced by the appearance of a second desorption feature at 197 °C. The effect may be associated with the creation of additional oxygen vacancies induced by CeO_2_ which have been reported to facilitate CO_2_ activation on ceria-containing catalysts [66]. A similar trend is observed for NiCeCa-MK, although the second weak desorption peak appears at a slightly lower temperature (175 °C). The result is in line with that obtained by Liang et al. [52]. In contrast, NiCeMg-MK exhibits only a single weak desorption feature centered at 102 °C, with no additional low-temperature desorption peak being detected. This observation suggests that CaO modifies the population of weak basic sites more effectively than MgO, although the origin of this difference may also involve differences in the interaction between alkaline earth oxides and ceria.

It has been reported that different basic sites may stabilize distinct reaction intermediates, including formate- and carbonate-type species, thereby influencing the CO_2_ methanation pathway [67]. In particular, medium-strength basic sites are considered the most favorable for formation of monodentate formate (HCOO^−^) species, a key intermediate in methane synthesis [68]. However, since no operando or in situ spectroscopic investigations were performed in the present study, the dominant reaction pathway cannot be conclusively identified. Nevertheless, the CO_2_-TPD results clearly demonstrate that the addition of MgO and CaO modifies the distribution and strength of basic sites, thereby enhancing CO_2_ adsorption and activation. Compared with Ni-MK, the addition of CeO_2_ substantially decreases the intensity of the medium-temperature desorption feature, although the desorption maximum occurs at a higher temperature (380 °C), indicating that fewer medium-strength basic sites are present, although they exhibit stronger CO_2_ binding. These desorption features are commonly attributed to carbonate species adsorbed on medium-strength metal–oxygen acid–base pairs. In the presence of ceria, Ce^4+^–O^2−^ pairs have been proposed as additional adsorption sites contributing to CO_2_ activation [69]. Moreover, the redox properties of the Ce^4+/^Ce^3+^ couple and the associated oxygen vacancies are considered crucial for CO_2_ adsorption. Battumur et al. [70] claimed that the oxygen vacancy is typically generated to compensate the imbalance of charge between Ni^2+^ and Ce^4+^ by creating metal–O^2−^ acid–base pairs, capable of CO_2_ dissociation I.

In contrast to CeO_2_ alone, the simultaneous addition of CeO_2_ and MgO generated an additional medium-temperature desorption peak at 435 °C while preserving the feature at ~330 °C, indicating a broader distribution of medium-strength basic sites. Thus, CeO_2_ strengthens CO_2_ adsorption, whereas MgO increases the population of medium-strength basic sites. Consistent with the H_2_-TPR results, Mg also modifies the interaction between the active phase and the support, thereby altering the surface properties of the catalyst [71]. In contrast, a high-temperature desorption peak at 560 °C was observed only for NiCeCa-MK, indicating the formation of strong basic sites, likely associated with additional oxygen vacancies induced by CaO [72]. These sites are expected to enhance CO_2_ chemisorption and may influence the methanation pathway, as discussed in the following section.

#### 2.1.6. Fourier-Transform Infrared Spectroscopy (FT-IR)

**Figure 12:**
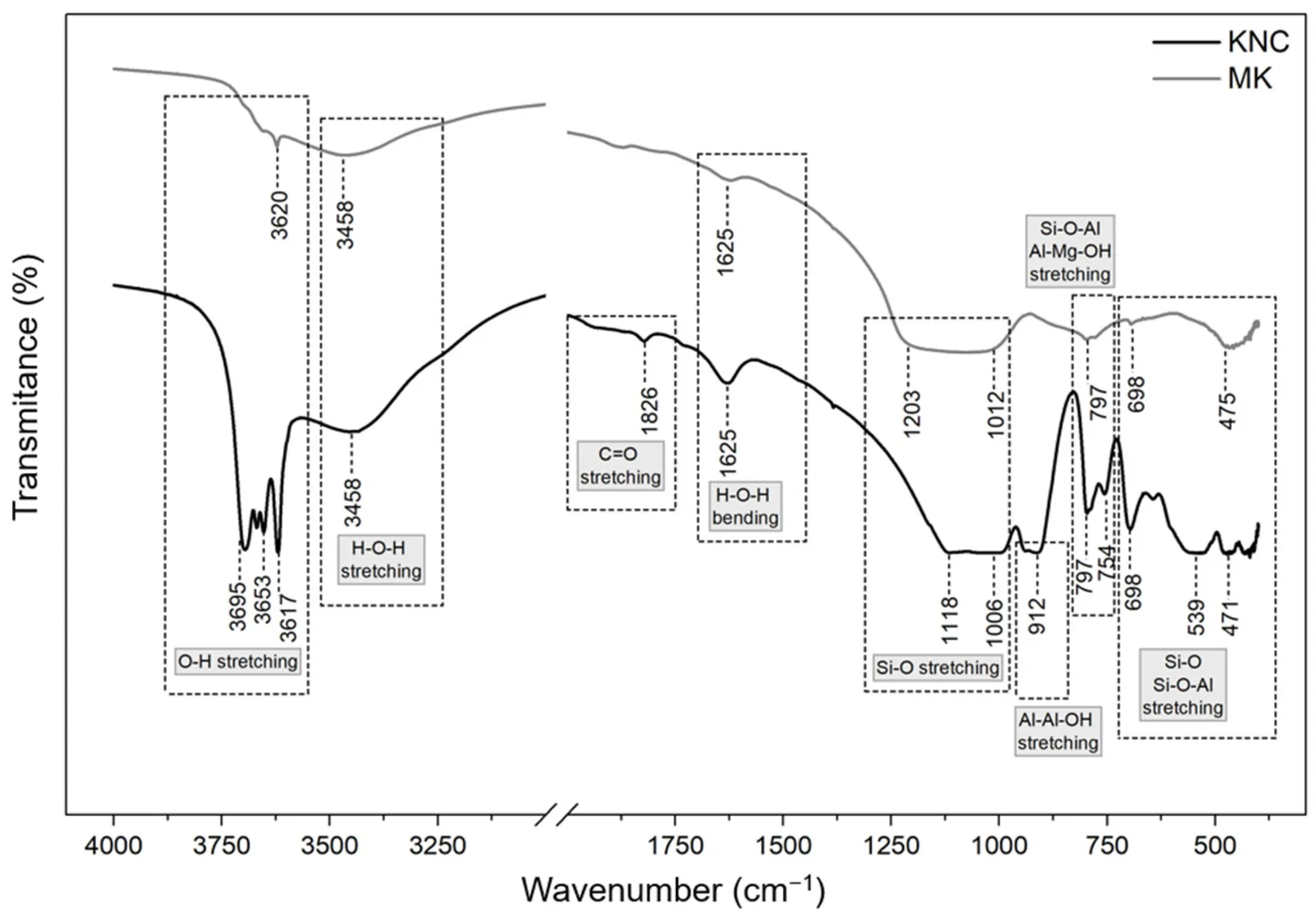
FT-IR spectra of kaolin (KNC) and metakaolin (MK).

FT-IR spectroscopy was used to investigate the structural changes occurring during the thermal transformation of kaolin. As shown in Figure 12**,** the spectra of kaolin and metakaolin exhibit characteristic aluminosilicate framework vibrations (1300–1400 cm^−1^) together with O-H stretching bands (4000–3000 cm^−1^) [73]. After calcination, the intensity of bands assigned to Al-OH, Si-O-Al, and structural water markedly decreased or disappeared, indicating dehydroxylation and partial amorphization of the kaolin structure. The medium-intensity peak at 1625 cm^−1^ appears due to H-O-H bending vibrations of the structural water molecules [43,74,75]. On the contrary to KNC, the spectrum recorded for MK exhibited limited number of low-intense absorption bands and the peak at 1625 cm^−1^ (H_2_O) was almost completely removed. These observations are consistent with the XRD and SEM results, confirming the disruption of the original layered crystal structure.

**Figure 13:**
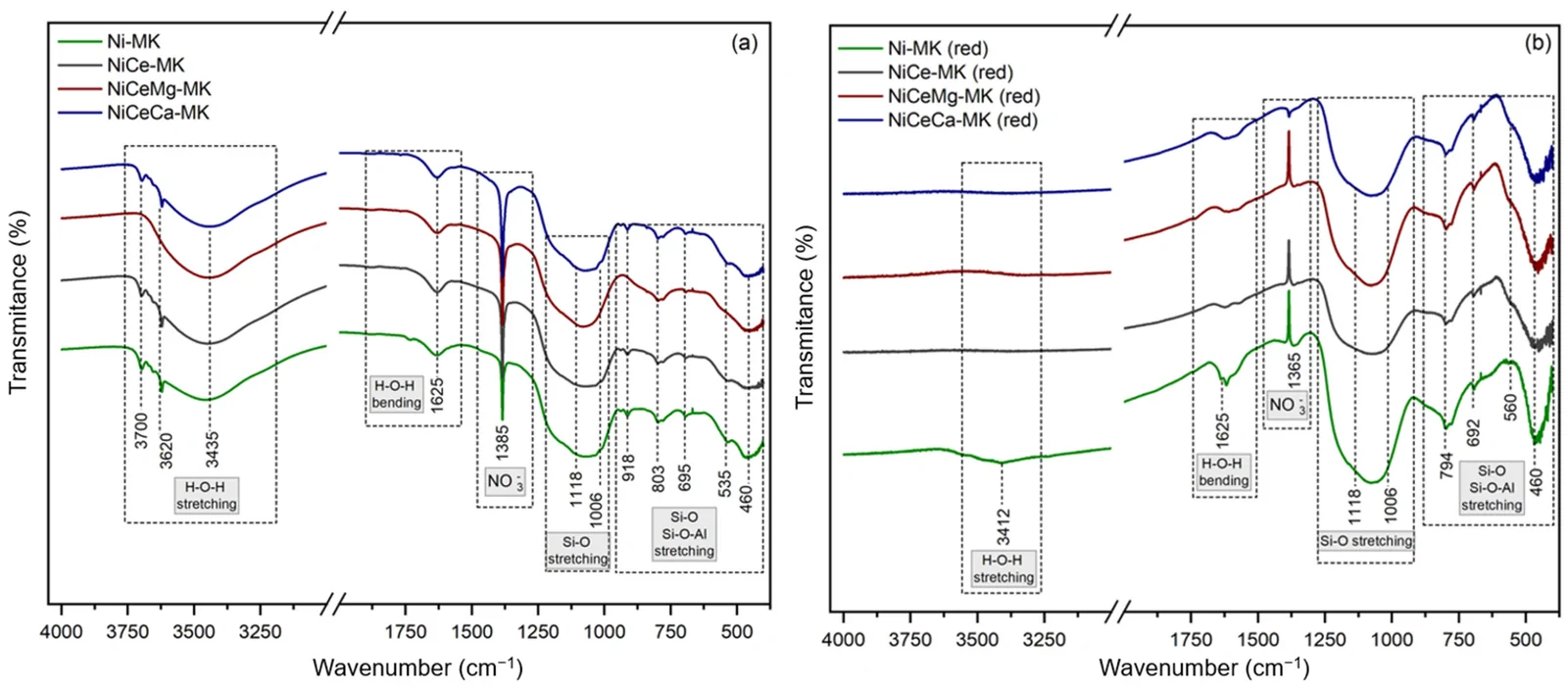
FT-IR spectra of (**a**) as-prepared catalysts and (**b**) reduced catalysts.

The FT-IR spectra of the as-prepared (Figure 13a) and reduced (Figure 13b) catalysts exhibited only minor differences. After H_2_ reduction, the broad O-H stretching bands (4000–3000 cm^−1^) almost completely disappeared, indicating the removal of residual water. The aluminosilicate framework vibrations (1300–400 cm^−1^) remained essentially unchanged, confirming that neither metal incorporation nor reduction affected the structure of the metakaolin support. A sharp band at 1385 cm^−1^, assigned to residual NO^3−^ species, was observed for the as-prepared catalysts, but nearly disappeared after reduction, indicating the removal of nitrate residues.

### 2.2. Results of CO_2_ Methanation Catalytic Tests

**Figure 14:**
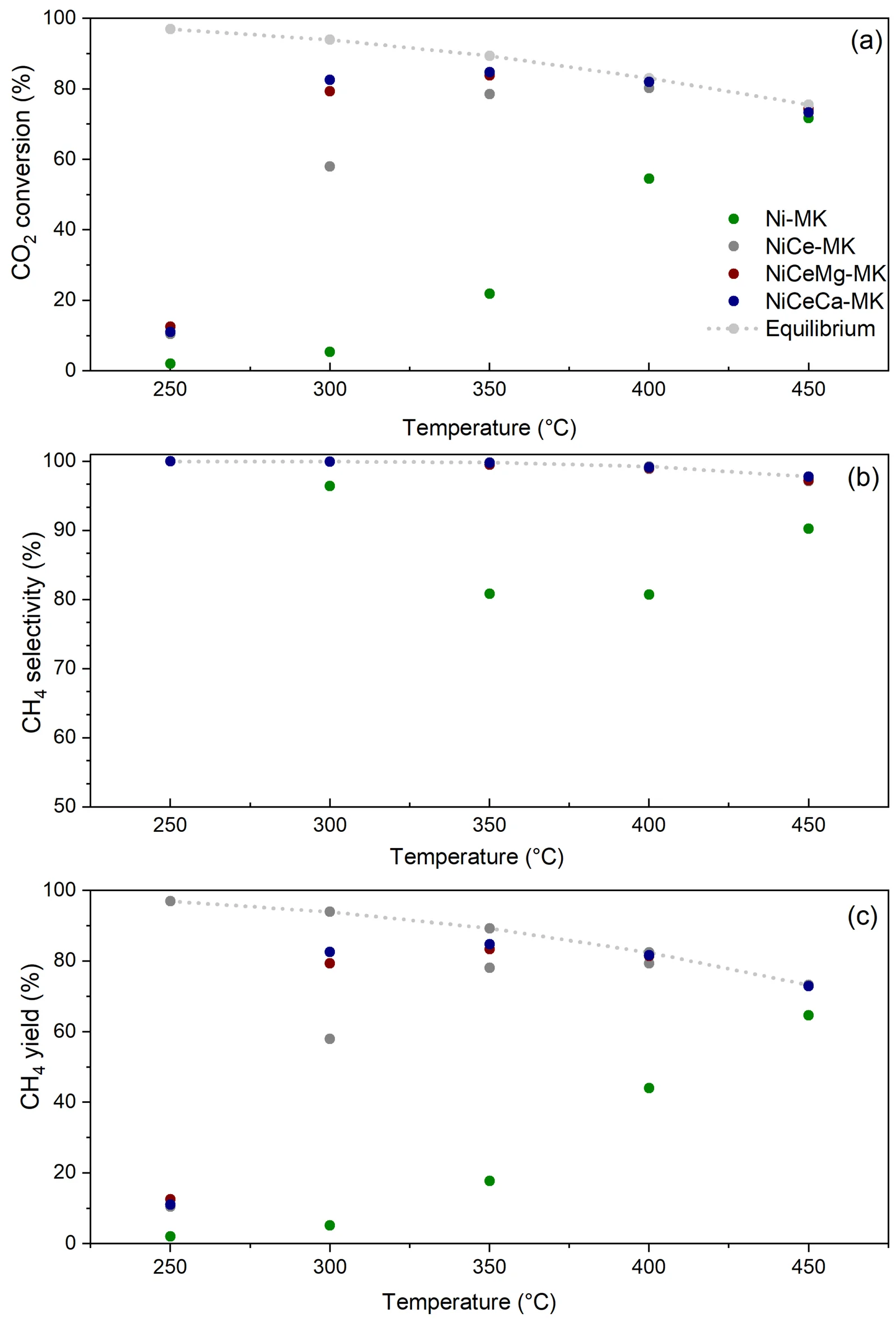
Results of CO_2_ methanation catalytic tests: (**a**) CO_2_ conversion; (**b**) selectivity to CH_4_; (**c**) CH_4_ yield.

Figure 14a shows CO_2_ conversion as a function of temperature for the investigated catalysts. The activity of all samples was observed to grow gradually with the increasing temperature. The highest CO_2_ conversion of approximately 80% was achieved at 400 °C for the promoted catalysts, approaching the thermodynamic equilibrium under the applied reaction conditions. Above this temperature, activity of all samples decreased due to the exothermic nature of methanation. The addition of CeO_2_ noticeably increased CO_2_ conversion at both low and high temperatures. This improvement can be attributed to lower CO_2_ dissociation energy provided by ceria [76], which facilitates CO_2_ activation [13,24,76]. Moreover, the improved performance may also result from the enhanced reducibility of NiO species and better textural properties, as evidenced by H_2_-TPR and N_2_ sorption results, respectively. Although CeO_2_ shifted the reduction features toward lower temperatures, indicating easier reduction of NiO, the Ni crystallite size after reduction remained nearly unchanged compared to Ni-MK (see Table 1). Therefore, the promotional effect of CeO_2_ cannot be explained solely by an increase in the metallic Ni surface area but should instead be attributed to the combined effects of improved reducibility of NiO and the enhanced CO_2_ activation provided by ceria.

Importantly, the incorporation of alkaline earth metal promoter significantly influenced catalytic performance, markedly increasing CO_2_ conversion. This behavior is likely associated with enhanced surface basicity, improved CO_2_ chemisorption capacity, and its activation induced by MgO and CaO [15]. Additionally, the Mg-promoted sample exhibited the highest specific surface area and total pore volume, which likely facilitated a more uniform distribution of nickel species across the catalyst surface. This observation is consistent with the EDS elemental mapping, which confirmed that the introduction of MgO enhances the dispersion of NiO and CeO_2_. Moreover, the H_2_-TPR profiles demonstrated that the simultaneous presence of CeO_2_ and MgO improved reducibility and weakened the metal–support interactions. The NiCeCa-MK sample exhibited almost identical catalytic performance as its Mg-promoted counterpart. However, its improved behavior cannot be assigned to structural–textural features nor reducibility. Based on SEM/EDS results, the promoting effect can be ascribed to uniform dispersion of the metallic phase in the presence of Ca. Furthermore, Xu et al. [60] reported that doping with calcium favored chemisorption of CO_2_ through the enhanced surface basicity. According to their results, the apparent CO_2_ activation energy decreased from 75.2 to 53.6 kJ/mol, improving the low-temperature catalytic activity. These results are in agreement with the outcomes of CO_2_-TPD, which indicated that strong basic sites were formed only in the case of NiCeCa-MK. Therefore, the positive impact of calcium can also be assigned to the modification of the chemical characteristics of the catalyst surface.

To further interpret the catalytic performance, the experimental results were analyzed in the context of thermodynamic limitations. As discussed by Wang et al. [77], the hydrogenation of CO_2_ is governed by both kinetic and thermodynamic factors, and the equilibrium methane yield remains high (>85%) at temperatures below 400 °C under near-atmospheric pressure for stoichiometric H_2_/CO_2_ mixtures. In the present study, the NiCeMg-MK and NiCeCa-MK catalysts exhibited CO_2_ conversions and CH_4_ selectivity approaching the corresponding equilibrium values, particularly at 350–400 °C. This suggests that the performance of these catalysts is approaching the thermodynamic limit, indicating that thermodynamic equilibrium rather than intrinsic catalytic activity becomes the dominant factor limiting further improvement. In contrast, the considerably lower CO_2_ conversion and CH_4_ selectivity observed for Ni-MK suggest that the methanation reaction remains kinetically limited. This distinction clearly demonstrates that the incorporation of Ce together with Mg or Ca not only enhances catalyst activity but also enables the methanation reaction to proceed close to the thermodynamic equilibrium under the applied reaction conditions. The lower CH_4_ selectivity further suggests that the subsequent hydrogenation of CO to CH_4_ is less effective, resulting in a greater contribution of the reverse water–gas shift reaction under the applied reaction conditions. Furthermore, the decrease in CO_2_ conversion observed at 450 °C follows the thermodynamic equilibrium trend, reflecting the progressive shift of the reaction equilibrium toward the reverse water–gas shift reaction at elevated temperatures, as predicted by thermodynamics. Despite this effect, the promoted catalysts maintained CH_4_ selectivity close to the equilibrium values throughout the investigated temperature range, confirming their high efficiency for CO_2_ methanation.

Overall, the results demonstrate that both Ca and Mg promoters effectively enhance catalytic activity. In contrast, Guo and Lu [14] confirmed that promotion with Ca only slightly altered the performance of Ni-SiO_2_; however, the addition of MgO lowered reducibility of Ni species, thus CO_2_ conversion. The effect was further confirmed by Méndez-Mateos et al. [11] who observed that incorporation of Ce as a single promoter improves Ni-based catalysts, whereas Ca doping has a negligible effect and Mg addition suppresses catalytic activity. Based on our study, the simultaneous deposition of Ce and an alkaline earth metal oxides enables the combination of the redox properties and basic character of CeO_2_, with the enhanced CO_2_ adsorption capacity provided by magnesium/calcium oxides. Furthermore, Liang et al. [52] reported that the addition of 5 wt.% Mg and Ca significantly increased the catalyst basicity, but also promoted the reverse water–gas shift reaction. In the present study, the same promoter loading (5 wt.%) was employed; however, enhanced CO_2_ methanation performance was observed. These differences suggest that the effect of alkaline earth promoters is governed not only by their loading, but also by the catalyst support and its interactions with the active phase and promoters. In our case, the metakaolin support (Al_2_O_3_ · 2 SiO_2_) appears to provide more favorable conditions for CO_2_ methanation than the γ-Al_2_O_3_ used by Liang et al.

As shown in Figure 14b, all catalysts except Ni-MK exhibited very high CH_4_ selectivity (>97%) over the entire investigated temperature range. In contrast, the lower CH_4_ selectivity observed for Ni-MK suggests a greater contribution of the reverse water–gas shift reaction, indicating that the subsequent hydrogenation of CO to CH_4_ was less effective over this catalyst [10]. Figure 14c presents the CH_4_ yield, which increased steadily with temperature for all samples, reflecting the combined effect of rising CO_2_ conversion and high CH_4_ selectivity.

**Figure 15:**
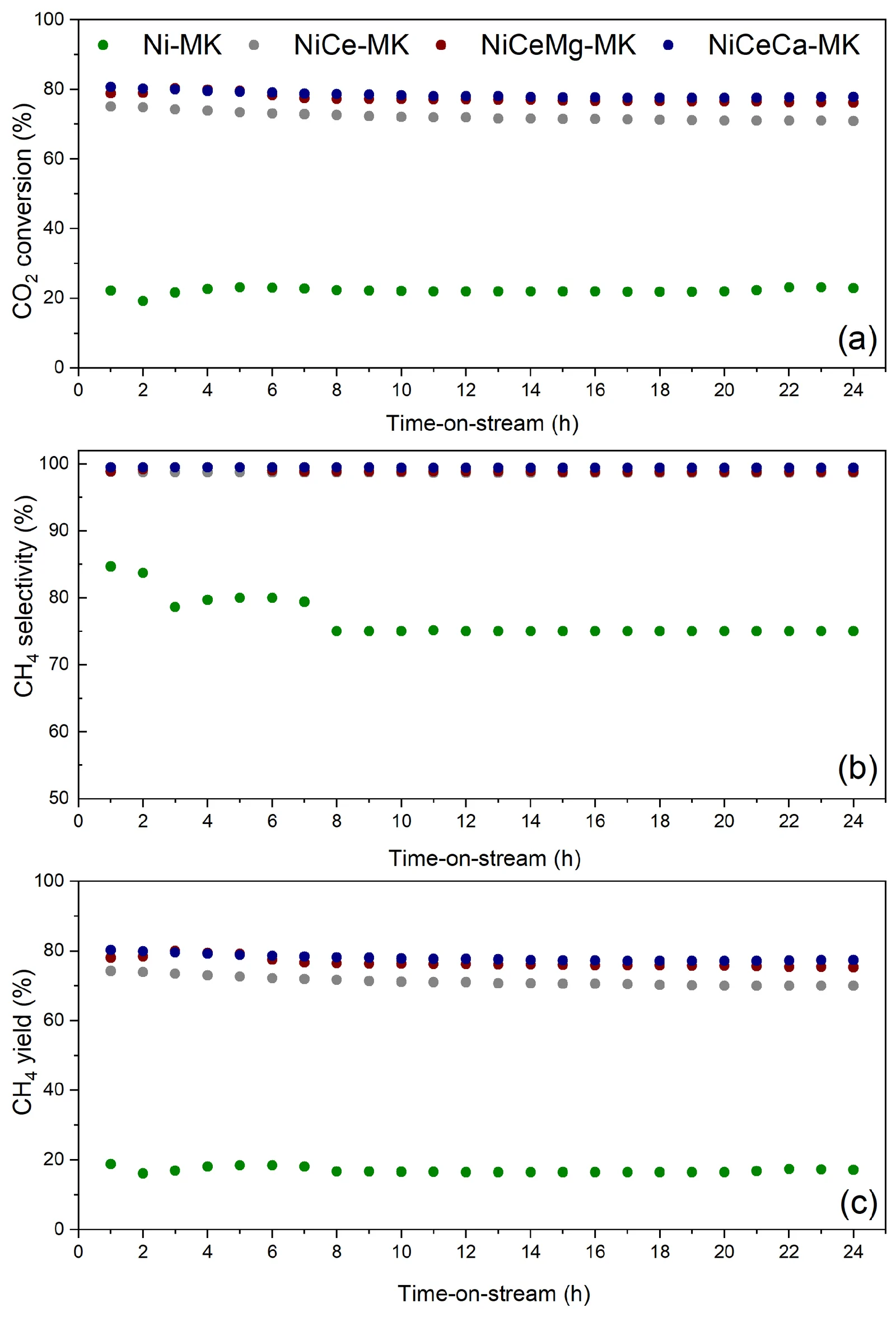
Time-on-stream stability tests during CO_2_ methanation: (**a**) CO_2_ conversion; (**b**) selectivity to CH_4_; (**c**) CH_4_ yield.

**Figure 16:**
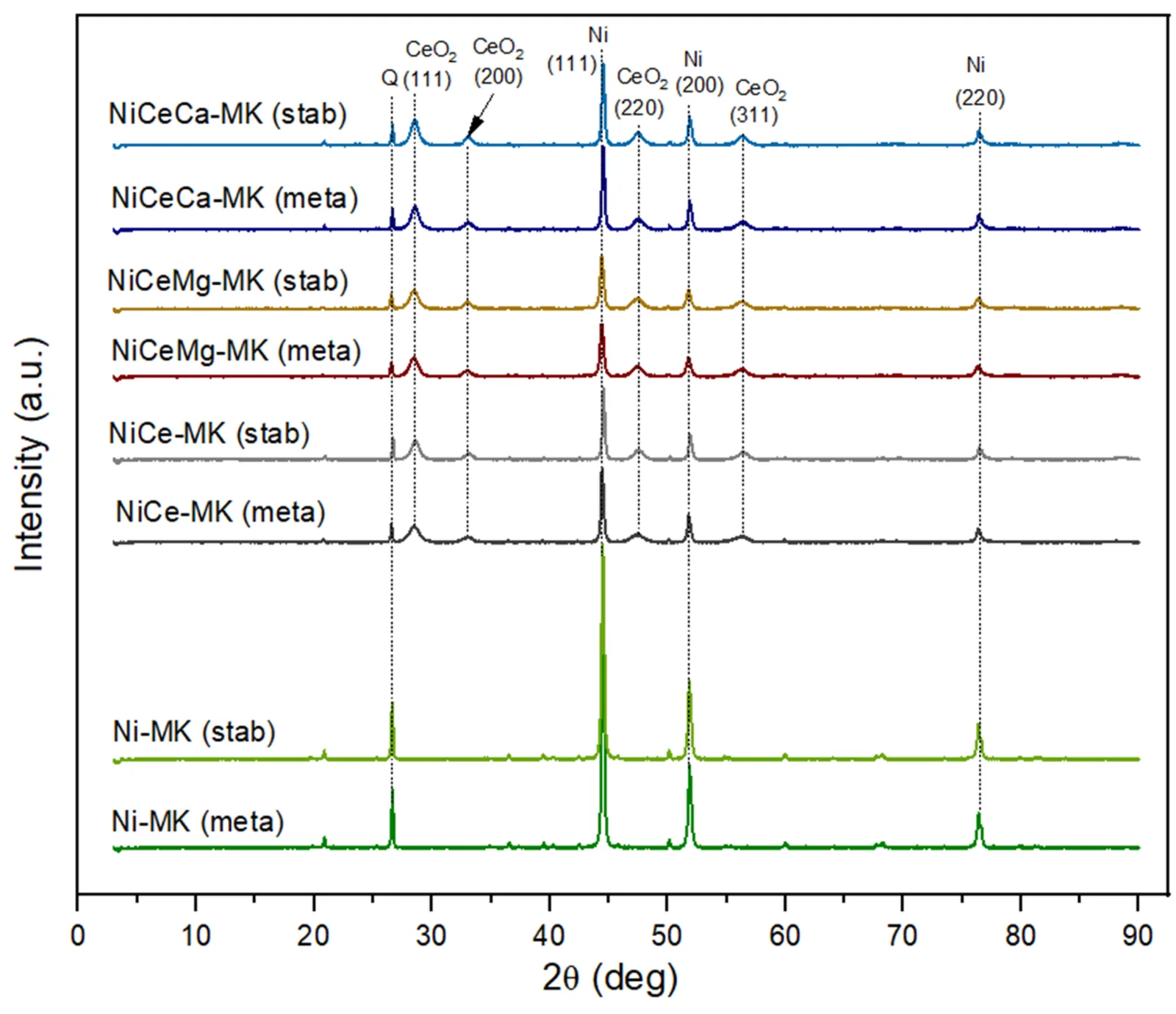
Comparison of the XRD patterns recorded for the materials after one catalytic cycle (meta) and stability tests (stab).

The time-on-stream stability tests, presented in Figure 15, were performed for the investigated samples at 350 °C for 24 h (GHSV of 12,000 h^−1^). The relatively low testing temperature of 350 °C could be applied because high CO_2_ conversion values had already been achieved at this temperature. The possible gradual deactivation of the catalysts was analyzed over time by observing changes in CO_2_ conversion, as well as CH_4_ selectivity and yield. Based on the obtained results, the activity and selectivity of all samples were maintained throughout the entire stability test. In the case of the Ni-MK catalyst, CO_2_ conversion increased during the first 3 h of the test and subsequently reached a stable level. This change may be related to the results presented in Table 1, which indicate a decrease in Ni crystallite size following the catalytic reaction. An increase in Ni crystallite size, as shown in Table 1, was observed exclusively for the NiCeCa-MK catalyst. Therefore, this sample may be considered less thermally stable. This observation is also supported by the results presented in Figure 15 where CO_2_ conversion decreased from 77.6% during the first hour of the test to 76.6% after 24 h. Importantly, the XRD analysis of the catalysts after the stability tests, presented in Figure 16, showed no detectable crystalline carbon phases, indicating no evidence of significant coke formation during the stability tests. Interestingly, although CeO_2_ has been reported to exhibit limited thermal stability under the highly exothermic conditions of CO_2_ methanation [78], the present results suggest that its combination with alkaline earth metal oxides enhances the resistance of the active phase to agglomeration.

## 3. Discussion

To elucidate the promoting effects of CeO_2_ and alkaline earth metal oxides (MgO and CaO), the structural, textural, redox, and surface properties of metakaolin-supported Ni catalysts were systematically correlated with their catalytic performance.

XRD confirmed that calcination of natural kaolin produced an amorphous metakaolin support. This transformation is essential, as dehydroxylation generates structural defects and coordinatively unsaturated Al centers that facilitate anchoring of the active phase and suppress its migration during thermal treatment [78,79]. Importantly, neither catalyst synthesis nor reduction induced recrystallization of the support. SEM observations further demonstrated that the characteristic plate-like morphology of metakaolin was preserved even after deposition of 30 wt.% Ni, confirming that the melt infiltration procedure does not deteriorate the aluminosilicate framework. Although CeO_2_ alone had little effect on the Ni^0^ crystallite size, its combination with MgO or CaO resulted in smaller Ni^0^ crystallites and a more homogeneous distribution of the metallic phase, as confirmed by SEM/EDS. These observations suggest that alkaline earth metal promoters suppress Ni agglomeration during catalyst preparation, consistent with previous reports on melt infiltration-derived catalysts. Cho et al. [80] applied this method to simultaneously impregnate γ-Al_2_O_3_ with Ni and selected alkaline earth metals, including Ca and Mg. The authors did not observe promotional effect of Mg on CO_2_ conversion. In contrast, the catalysts investigated in the present study, containing 5 wt.% Mg, exhibited improved catalytic performance, suggesting that the effect of Mg promotion depends not only on the promoter loading, but also on the catalyst composition, support properties, and preparation method. The lack of a positive impact of Mg reported by Cho et al. was attributed to the incomplete reduction of NiO and, consequently, to an insufficient amount of active metallic Ni^0^ species. Moreover, the authors compared melt infiltration with dry and wet impregnation methods, confirming that the former procedure gives the best catalytic results. In their alternative study on the application of melt infiltration in catalysts synthesis [81], it was confirmed that one of its most important advantages is the formation of well-dispersed active sites. Nitrogen physisorption further confirmed that after thermal activation, the support evolved from a fine-pore layered clay into a predominantly mesoporous material offering improved accessibility for the metallic phase. After catalyst preparation, the textural properties became strongly dependent on promoter composition. CeO_2_ increased the BET surface area and preserved mesoporosity, in agreement with previous reports describing ceria as an effective inhibitor of pore blockage by NiO species [13,48]. The most pronounced effect was observed after simultaneous incorporation of MgO, resulting in the highest specific surface area and total pore volume among all investigated catalysts. In contrast, CaO slightly reduced the surface area and pore volume, most likely because of partial pore occupation by the metallic phase and/or limited formation of calcium aluminate species, as previously reported.

H_2_-TPR further clarified the role of the promoters. The addition of CeO_2_ shifted the reduction features toward lower temperatures, confirming facilitated NiO reduction. This effect is commonly attributed to the high oxygen mobility of ceria and hydrogen spillover at the Ni–CeO_2_ interface, which promotes oxygen removal from NiO [82,83]. Since CeO_2_ had little effect on the Ni crystallite size, its promotional role is more likely associated with enhanced reducibility and oxygen transfer than with an increased metallic Ni surface area. The simultaneous addition of MgO further improved catalyst properties. Besides exhibiting the highest specific surface area, NiCeMg-MK showed the lowest low-temperature reduction peak, indicating readily reducible Ni species. In contrast, the superior performance of NiCeCa-MK appears to originate from a different mechanism. Although its textural properties and reducibility were inferior to those of NiCeMg-MK, SEM/EDS confirmed similarly uniform metal dispersion, while CO_2_-TPD revealed the formation of stronger basic sites. These results suggest that Ca primarily enhances CO_2_ adsorption and activation rather than modifying the metallic phase.

These structure–property relationships are directly reflected in the catalytic performance. All catalysts approached thermodynamic equilibrium near 400 °C, whereas CO_2_ conversion decreased at higher temperatures due to the exothermic nature of the Sabatier reaction. The addition of CeO_2_ enhanced CO_2_ conversion across the entire temperature range despite having little effect on the Ni^0^ crystallite size, indicating that its promotional effect is primarily associated with improved reducibility and oxygen mobility. An even greater improvement was achieved by the simultaneous incorporation of MgO or CaO, particularly at 300–350 °C. In agreement with the physicochemical characterization, the enhanced activity of NiCeMg-MK is attributed to improved textural properties, Ni dispersion, and reducibility, whereas the superior performance of NiCeCa-MK mainly results from increased surface basicity and enhanced CO_2_ adsorption.

The CO_2_-TPD results further confirm that the selected promoter loading (5 wt.%) effectively strengthened surface basicity without excessively blocking active nickel sites. As evidenced, the introduction of MgO or CaO noticeably enhanced the conversion of carbon dioxide compared to Ni-MK and NiCe-MK, particularly at 300 °C. This effect might be ascribed not only to the structural characterization of the samples, but also to their surface basicity, which was strengthened after modification with alkaline earth oxides. According to the investigation carried out by Xu et al. [15], promotion of nickel methanation catalysts with Mg intensifies chemisorption and subsequent activation of CO_2_, which ultimately decreases the activation energy of the process. However, the authors also observed the so-called “volcano effect” related to the strong correlation between the loading of magnesium and its beneficial effect on catalytic performance. In general, it was observed that the excess of the basic promotor firmly adsorbs carbon dioxide, with simultaneous suppression of the desorption step. In consequence, the adjacent nickel active species become covered and blocked against activation of hydrogen. The authors also observed this relationship while promoting Ni-catalysts with Ca [60]. The selected loading of alkaline earth metal promoters proved to be effective, as the promoted catalysts approached thermodynamic equilibrium with increasing temperature. An additional advantage of the proposed approach is the use of melt infiltration, which enabled homogeneous incorporation of 30 wt.% Ni while preserving the metakaolin framework and preventing the formation of large Ni agglomerates. Consequently, the catalysts combined high Ni loading with excellent metal dispersion, which is difficult to achieve using conventional synthesis methods.

Besides activity, catalyst durability is crucial for practical CO_2_ methanation. Stability tests performed at 350 °C showed that all catalysts maintained high CH_4_ selectivity throughout 24 h on stream, while the promoted samples exhibited only minor changes in CO_2_ conversion, indicating high resistance to thermal deactivation. XRD analysis of the spent catalysts revealed no detectable crystalline carbon phases. Although NiCeCa-MK showed a slight increase in Ni crystallite size accompanied by a small decrease in CO_2_ conversion, the changes remained limited, confirming that the combined use of CeO_2_ and alkaline earth promoters suppresses Ni agglomeration during prolonged operation.

Overall, the results demonstrate that metakaolin is a sustainable and effective support for CO_2_ methanation catalysts owing to its thermal stability and ability to accommodate high Ni loadings. Combined with melt infiltration, it enables the preparation of well-dispersed catalysts with high reducibility, excellent CH_4_ selectivity, and long-term stability. These findings highlight the potential of clay-derived materials as sustainable alternatives to conventional oxide supports for CO_2_ valorization.

## 4. Materials and Methods

### 4.1. Preparation of the Catalysts

Amorphous metakaolin was obtained by calcination of natural kaolin (white kaolinite clay, Calaya) in air at 550 °C for 4 h, using a heating rate of 2 °C/min. Catalyst samples were prepared by a one-step melt infiltration method. Prior to metal incorporation, the metakaolin support was finely ground in a ceramic mortar. Nickel(II) nitrate hexahydrate (Ni(NO_3_)_2_ · 6H_2_O), cerium(III) nitrate hexahydrate (Ce(NO_3_)_3_ · 6H_2_O), magnesium nitrate hexahydrate (Mg(NO_3_)_2_ · 6H_2_O), and calcium nitrate tetrahydrate (Ca(NO_3_)_2_ · 4H_2_O) were used as metal precursors. Appropriate amounts of metal hydrate precursors were then added to achieve metal loadings of 30 wt.% Ni, 15 wt.% Ce, and 5 wt.% Mg or 5 wt.% Ca, with the loadings calculated on an elemental basis. The amounts of the precursor salts were calculated to yield 5 g of each catalyst. The precursor salts were mixed with the metakaolin support and thoroughly ground in a ceramic mortar until a homogeneous solid mixture was obtained. During the subsequent thermal treatment, the hydrated nitrate salts gradually melted and infiltrated the metakaolin matrix, followed by their thermal decomposition to the corresponding oxide species, resulting in a uniform distribution of the active components within the support. The resulting powders were transferred to ceramic crucibles and subjected to a controlled stepwise thermal treatment. The samples were heated at a rate of 1.25 °C/min to 100 °C and held for 2 h, followed by heating at 1.6 °C/min with an additional dwell at 100 °C for 2 h. Subsequently, the temperature was increased to 200 °C at 1.6 °C/min and maintained for 3 h. In the final step, the samples were heated to 350 °C at a ramp rate of 1.25 °C/min and held for 3 h. The codes of the samples with the corresponding descriptions are collected in Table 5.

### 4.2. Characterization of the Catalysts

The physicochemical properties of the supports and the catalysts were examined using a combination of characterization techniques. Structural ordering and phase composition were assessed by X-ray diffraction (XRD) performed on a Panalytical Empyrean diffractometer, employing Cu K*α* radiation (λ = 1.5406 Å) in Bragg–Brentano configuration. Diffraction patterns were collected over a 2*θ* range of 3–90° for the as-synthesized and reduced samples, and the average crystallite size of Ni^0^ was estimated using the Scherrer Equation (1), after correcting for instrumental broadening and using a shape factor of 0.89 [80]:

(1) $$D=\frac{k\cdot \lambda }{\beta \cdot cos\theta }$$

where *k*—shape factor, *β*—full width at half maximum of the peak (FWHM), *λ*—X-ray wavelength, *θ*—diffraction angle (in radians).

Textural properties were determined from N_2_ adsorption–desorption isotherms collected at 77 K using a Micromeritics gas adsorption analyzer equipped with 3Flex Surface Characterization software, version 5.03 (Micromeritics Instrument Corporation, Norcross, GA, USA). Before the measurement, the samples were degassed under vacuum at 30 °C for 1 h, 90 °C for 1 h, 200 °C for 6 h, and 300 °C for 3 h. The BET (Brunauer–Emmett–Teller) method was applied to calculate the specific surface area (*S_BET_*), while micropore volume and external surface area were obtained from t-plot analysis. Mesopore size distributions were derived using the BJH model from the desorption branch. Morphology and element distributions in the samples were analyzed by SEM-EDS on a JEOL JSM-7500F microscope (JEOL, Tokyo, Japan) equipped with an AZtecLiveLite Xplore 30 EDS system (Oxford Instruments NanoAnalysis, High Wycombe, UK). Prior to the analysis, the powdered samples were deposited on a carbon adhesive tape mounted on aluminum stubs and sputter-coated with chromium to ensure electrical conductivity for the analysis. Redox properties were evaluated by temperature-programmed reduction with hydrogen (H_2_-TPR) by a Micromeritics AutoChem III chemisorption analyzer software version 1.00 (Micromeritics Instrument Corporation, Norcross, GA, USA). During the measurements, the catalysts were heated from room temperature to 600 °C at a rate of 10 °C min^−1^ under a 50 cm^3^/min flow of a gas mixture containing 5 vol.% H_2_ in Ar. The amount of consumed H_2_ was quantified after baseline correction using Gaussian fitting and deconvolution of the reduction profiles. Thermo-programmed CO_2_ sorption/desorption experiments were performed using AMI-300 (Altamira Instruments, Cumming, GA, USA) Chemisorption Analyzer equipped with Thermal Conductivity Detector (TCD) detector. The sample (ca. 0.05 g) was placed inside a quartz U-tube that was set in the oven of the Altamira AMI-300. Helium was applied as an inert gas while 5 vol.% H_2_/Ar was applied as the reductive gas. For the sorption gas, 5% vol. CO_2_ in Ar was used. Thermal desorption was performed in He flow. The sample was activated in the flow of He. First, the sample was held at 25 °C (He 30 cm^3^/min) for 80 min, then heated to 120 °C (heating rate of 10 °C/min, He flow of 30 cm^3^/min) and held at 120 °C for 60 min (He 30 cm^3^/min), and cooled down to 50 °C (He cm^3^/min). The sample was reduced in the flow of 5 vol. % H_2_ in Ar (30 cm^3^/min) at 45–50 °C for 60 min. Next, the sample was held in He flow (30 cm^3^/min) at 50 °C ca. 30 min. The CO_2_ sorption was performed at 50 °C in the flow of 5 vol.% of CO_2_ in Ar (30 cm^3^/min). After the CO_2_ sorption, the sample was kept in He flow (30 cm^3^/min) at 50 °C for 30 min. When the stable intensity of CO_2_ line (*m*/*z* = 44) was observed, CO_2_ desorption was performed under heating from 50 to 650 °C (heating rate of 10 °C/min, He 30 cm^3^/min). The surface functionalities were probed by ATR FT-IR spectroscopy with a Perkin Elmer Frontier spectrometer (PerkinElmer, Waltham, MA, USA) over the range of 4000–650 cm^−1^. Comparing the spectra before and after reduction provided insights into the stability of the aluminosilicate framework and the evolution of the coordination environment of nickel species.

### 4.3. CO_2_ Methanation Catalytic Tests

CO_2_ methanation experiments were carried out in a continuous-flow fixed-bed quartz microreactor operated at atmospheric pressure. The reaction temperature was monitored and controlled by a K-type thermocouple positioned directly in the catalytic bed to ensure accurate measurement of the real working temperature. Prior to testing, all catalysts were subjected to an activation step consisting of reduction under a 5 vol.% H_2_/Ar mixture (100 cm^3^/min). The samples were heated from room temperature to 600 °C at a rate of 10 °C/min. The final temperature was maintained for 1 h, enabling efficient conversion of NiO to metallic Ni, while preventing undesirable structural effects associated with higher temperatures. Immediately after the reduction process, the reactor was switched to the reaction feed, without exposing the catalysts to ambient conditions. The reactant stream consisted of CO_2_/H_2_/Ar with a molar ratio of 1.5/6/2.5, and the total flow was maintained at 100 cm^3^/min, corresponding to a gas hourly space velocity (GHSV) of 12,000 h^−1^. Reactor outlet gases were continuously analyzed by an ABB EL3020 on-line FT-IR analyzer (ABB Automation GmbH, Frankfurt am Main, Germany) for CH_4_ and CO, whereas the residual CO_2_ fraction was quantified using a Hartmann & Braun Advance Optima gas analyzer (ABB Automation GmbH, Frankfurt am Main, Germany). Catalytic activity measurements were carried out in the temperature range of 250–450 °C in a stepwise mode, allowing 30 min at each temperature to ensure steady-state operation. The heating rate between the temperature steps was 10 °C/min. Long-term stability tests were performed under the same flow composition and GHSV as the standard activity measurements. The most active catalysts were operated at their respective optimal temperatures for 24 h to evaluate possible deactivation phenomena. Theoretical equilibrium values of CO_2_ conversion and CH_4_ selectivity were calculated using the DWSIM Simulator. The conversion of CO_2_ $\left(X_{{CO}_{2}}\right)$ and selectivity to CH_4_ ($S_{{CH}_{4}}$) were calculated according to Equations (2) and (3), respectively:

(2) $$X_{{CO}_{2}}=\frac{F_{{CO}_{2},in}-F_{{CO}_{2},out}}{F_{{CO}_{2},in}}\cdot 100\%$$

(3) $$S_{{CH}_{4}}=\frac{F_{{CH}_{4}}}{F_{{CH}_{4}}+F_{CO}}\cdot 100\%$$

where *F* corresponds to the flow calculated from the concentration of gases.

Methane yield ($Y_{{CH}_{4}})$ resulted from the combination of CO_2_ conversion and selectivity to CH_4_ (4):

(4) $$Y_{{CH}_{4}}=\frac{X_{{CO}_{2}}\cdot S_{{CH}_{4}}}{100\%}$$

## 5. Conclusions

Metakaolin derived from natural kaolin proved to be an effective support for Ni-based CO_2_ methanation catalysts prepared using the melt infiltration method. Thermal activation produced an amorphous aluminosilicate support capable of accommodating high Ni loadings without structural degradation. The addition of CeO_2_ improved NiO reducibility, while MgO and CaO enhanced catalyst texture, surface basicity, and active phase dispersion, resulting in superior catalytic performance. Consequently, the Ce- and alkaline earth-promoted catalysts exhibited higher CO_2_ conversion than Ni-MK and NiCe-MK, while maintaining excellent CH_4_ selectivity (>97%) over the investigated temperature range. Stability tests confirmed high resistance to deactivation, with only minor changes in Ni crystallite size and no detectable crystalline carbon phases after reaction. Overall, the results establish clear structure–performance relationships and demonstrate that the combination of CeO_2_ with small amounts of MgO or CaO is an effective strategy for designing highly active and stable CO_2_ methanation catalysts. Furthermore, the study highlights metakaolin as a sustainable catalyst support and melt infiltration as a simple and scalable preparation method for high-loading Ni catalysts.

## Figures and Tables

**Table 1:** Crystallite size of NiO (as-prepared catalysts) and Ni (reduced, spent catalysts) calculated based on XRD results.

Sample	NiO, As-Prepared Catalyst (nm)	CeO_2_, As-Prepared Catalyst (nm)	Ni, Reduced Catalyst (nm)	Ni, Spent Catalyst ^[a]^ (nm)	Ni, Spent Catalyst ^[b]^ (nm)
Ni-MK	24	-	29	33	32
NiCe-MK	20	7	28	31	34
NiCeMg-MK	17	6	24	22	24
NiCeCa-MK	19	7	25	32	30

^[a]^ Ni crystallite size after one catalytic cycle. ^[b]^ Ni crystallite size after 24 h time-on-stream test at 350°C.

**Table 2:** Structural and textural properties of the materials.

Sample	Specific Surface Area ^[a]^ (m^2^/g)	External Surface Area ^[b]^ (m^2^/g)	Micropore Area ^[b]^ (m^2^/g)	Micropore Volume ^[b]^ (cm^3^/g)	Total Pore Volume ^[c]^ (cm^3^/g)	Average Pore Diameter ^[d]^ (nm)
KNC	15	13	2	0.0011	0.11	30
MK	10	9	1	0.1237	0.12	49
Ni-MK	23	19	4	0.0019	0.06	10
NiCe-MK	37	29	8	0.0032	0.10	11
NiCeMg-MK	39	30	9	0.0046	0.17	14
NiCeCa-MK	19	16	3	0.0012	0.02	11

^[a]^ Specific surface area determined by the BET method. ^[b]^ External surface area and micropore area obtained from the t-plot method. ^[c]^ Total pore volume calculated using BJH method. ^[d]^ Average pore diameter estimated from BET data using the 4V/A approximation.

**Table 3:** Chemical composition of the samples determined by EDS.

Sample	Si (wt.%)	Al (wt.%)	O (wt.%)	Ni (wt.%)	Ce (wt.%)	Mg (wt.%)	Ca (wt.%)
MK	26.3	21.7	52.0	-	-	-	-
Ni-MK	12.9	13.2	40.1	33.7	-	-	-
NiCe-MK	12.2	8.7	32.4	34.7	12.1	-	-
NiCeMg-MK	6.4	7.7	36.7	30.3	14.1	4.9	-
NiCeCa-MK	5.1	5.9	27.2	36.9	20.8	-	4.1

**Table 4:** Hydrogen consumption determined by H_2_-TPR analysis for Ni-based catalysts.

Sample	Low Temperature Hydrogen Uptake ^[a]^ (%)	High Temperature Hydrogen Uptake ^[b]^ (%)	Total Hydrogen Uptake (mmol/g)
Ni-MK	5.3	94.7	5.23
NiCe-MK	3.4	96.6	4.49
NiCeMg-MK	3.7	96.3	4.15
NiCeCa-MK	8.2	91.8	5.16

^[a]^ The low-temperature region: the reduction of weakly interacting NiO species, ≤250 °C. ^[b]^ The high-temperature region: the reduction of strongly interacting NiO species, >250 °C.

**Table 5:** List of samples with their codes and descriptions.

Sample Code	Description
Ni-MK	30 wt.% of Ni on metakaolin
NiCe-MK	30 wt.% of Ni, 15 wt.% Ce on metakaolin
NiCeMg-MK	30 wt.% of Ni, 15 wt.% Ce, 5 wt.% Mg on metakaolin
NiCeCa-MK	30 wt.% of Ni, 15 wt.% Ce, 5 wt.% Ca on metakaolin

## Data Availability

The original contributions presented in this study are included in the article. Further inquiries can be directed to the corresponding author.

## Abbreviations

The following abbreviations are used in this manuscript:

CCUS	Carbon Capture, Utilization and Storage
CO_2_-TPD	Carbon dioxide temperature-programmed desorption
FT-IR	Fourier-transform infrared spectroscopy
H_2_-TPR	Hydrogen temperature-programmed reduction
IUPAC	International Union of Pure and Applied Chemistry
JCPDS	Joint Committee on Powder Diffraction Standards
KNC	Non-calcined kaolin
MK	Metakaolin
PSD	Pore size distribution
RWGS	Reverse water–gas shift
*S_BET_*	Brunauer–Emmett–Teller specific surface area
SEM/EDS	Scanning electron microscopy coupled with energy-dispersive X-ray spectroscopy
*S_ext_*	External surface area
*S_micro_*	Micropore surface area
*V_micro_*	Micropore volume
*V_total_*	Total pore volume
XRD	X-ray diffraction
